# Sharks, rays and skates (Chondrichthyes, Elasmobranchii) from the Upper Marine Molasse (middle Burdigalian, early Miocene) of the Simssee area (Bavaria, Germany), with comments on palaeogeographic and ecological patterns

**DOI:** 10.1007/s12542-020-00518-7

**Published:** 2020-06-02

**Authors:** Jaime A. Villafaña, Giuseppe Marramà, Stefanie Klug, Jürgen Pollerspöck, Markus Balsberger, Marcelo Rivadeneira, Jürgen Kriwet

**Affiliations:** 1 Department of Palaeontology, University of Vienna, Althanstraße 14, 1090 Vienna, Austria; 2 Centro de Investigación en Recursos Naturales y Sustentabilidad, Universidad Bernardo O’Higgins, Santiago, Chile; 3 School of Science (GAUSS), Georg–August University, 37077 Göttingen, Germany; 4 Bavarian State Collection of Zoology, Munich, Germany; 5 Achberg 11, 83259 Schleching, Germany; 6 Centro de Estudios Avanzados en Zonas Áridas, Av. Ossandon 877, Coquimbo, Chile; 7 Departamento de Biología Marina, Facultad de Ciencias del Mar, Universidad Católica del Norte, Coquimbo, Chile; 8 Departamento de Biología, Universidad de La Serena, La Serena, Chile; 9 Dipartimento di Scienze della Terra, Università degli Studi di Torino, Via Valperga Caluso, 35, 10125 Torino, Italy

**Keywords:** Chondrichthyes, Molasse, Burdigalian, Early Miocene, Paratethys, Beta diversity

## Abstract

Elasmobranch remains are quite common in Miocene deposits and were the subject of numerous studies since the middle of the nineteenth century. Nevertheless, the taxonomic diversity of the Marine Molasse sharks, rays and skates is still largely unknown. Here, we describe 37 taxa from the lower Miocene of the Molasse Basin: 21 taxa could be identified at species level, whereas 15 taxa could only be assigned to genus and one taxon is left as order incertae sedis. The material was collected from deposits of the Auwiesholz Member of the Achen Formation (middle Burdigalian, middle Ottnangian age, ca. 17.8 Ma) exposed near Simssee, Upper Bavaria. This faunal assemblage is a mixture of shallow marine, near-coastal, pelagic and deep-water taxa. The fauna from Simssee displays different biogeographic dynamics at local and regional scales, possibly related to the intense climatic, oceanographic and tectonic events that occurred during the Eggenburgian–Ottnangian stages. The faunal relationships of the early Miocene chondrichthyan faunas from the Mediterranean Sea and Paratethys with others regions are established on the basis of qualitative (presence/absence) data. The beta diversity (Sørensen–Dice coefficient) of the Miocene Molasse elasmobranchs was used to characterize the taxonomic differentiation between localities and regions. According to our results, the fauna from Simssee shows close similarities with those from Switzerland, Austria, France and northern Germany. Faunal similarities and differences are mainly related to tectonic events and oceanographic variables (i.e. migration through seaway passages) or might represent collecting biases.

## Introduction

After the Tethys Ocean had nearly completely vanished by the end of the Eocene, an isolated Paratethys Sea developed in the latest Eocene–earliest Oligocene related to the development of the Alpine mountains (Baldi 1980; Rusu 1988; Rögl 1999). This island chain acted as barrier partly separating the Paratethys from the Mediterranean Sea. The Paratethys extended from the Rhone Valley in the east towards Inner Asia. Marginal to the Paratethys, the Molasse Basin, which represents a foreland basin, developed in the Oligo–Miocene during the Alpine–Himalayan orogeny. The Molasse Basin of southern Germany was thus part of western Paratethys during the Miocene.

The Paratethys and Mediterranean seas experienced dramatic changes during their development (Rögl 1999; Piller et al. 2007; Sant et al. 2017). During the Eggenburgian (lower Burdigalian), a broad sea passage between the Paratethys Sea and the Indo-Pacific Ocean was open, providing optimal environmental conditions for marine faunas and opportunities for widespread faunal exchanges (Rögl 1999). Additionally, a seaway passage through the Alpine fore-deep between the Mediterranean and Paratethys was open. Later, during the Ottnangian (middle Burdigalian), the sea passage into the Indo-Pacific Ocean was closed due the collision of Africa and Arabia with the Anatolian plate. The connection between the Western–Central Paratethys and the Mediterranean seas still persisted through the Rhine Graben, but the eastern Paratethys was already isolated (i.e. it informed the so-called Kotsakhurian Sea). All these events also induced changes in sea levels, salinity and temperature (Haq et al. 1988). Studies based on early Miocene marine invertebrates of Europe indicate that these intense climatic and oceanographic events had important effects on diversification patterns of organisms (Kroh 2007).

Remains of sharks, rays and skates generally are quite common in Miocene sediments of the Paratethys (Barthelt et al. 1991; Kocsis 2007; Reinecke et al. 2011; Schultz 2013; Pollerspöck and Straube 2017; Szabó and Kocsis 2016; Underwood and Schlögl 2013), but despite all progress accomplished in the last decades, our understanding of Miocene elasmobranchs taxonomic diversities and faunal relationships remains very incomplete. Here, we document an elasmobranch assemblage from the lower Miocene Upper Marine Molasse of the western Paratethys and present results about their relationships with other faunas from the early Miocene.

## Materials and methods

### Data collection

**Fig. 1:**
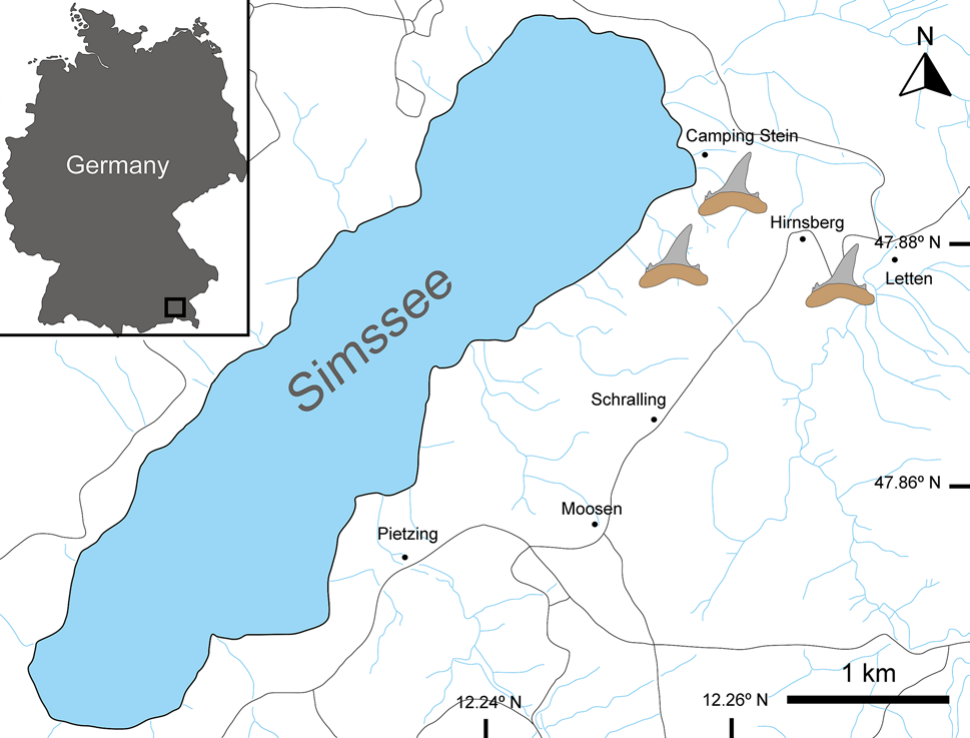
Map of the Simssee area

A total of 466 elasmobranch specimens (including teeth and tail spines) were recovered by screen washing and surface collecting from several points along the Auwiesholz Member of the Achen Formation in the Simssee area (Bavaria, S. Germany, Fig. 1), during several trips conducted by two of the authors (JP and MB) and other collaborators in the late 90s. Part of the material was collected by N. Rückert-Ülkümen (Bavarian State Collection of Palaeontology and Geology) near the village of Hirnsberg in 1993. The precise stratigraphic origin of all material, however, remains ambiguous. The Achen Formation is of middle Ottnangian age (middle Burdigalian, early Miocene, ca. 17.8 Ma) and denotes the second cycle of the Upper Marine Molasse in Bavaria (Pippèrr et al. 2007). The depositional setting represents an inner neritic environment basally, which deepens upwards. This interpretation was based on the presence of foraminifera (e.g. *Ammonia*) and ostracoda (e.g. *Cytheretta*), and vertebrates as bony fishes (e.g. *Rhynchoconger*) were also reported from this member. The elasmobranch fossil fauna described herein is almost coeval to the assemblage from the Baltringer Horizon in Baden-Württemberg, SW Germany (Probst 1879).

The fossils are housed in the Bayerische Staatssammlung für Paläontologie und Geologie at Munich, Germany, with the catalogue numbers bearing the prefix SNSB-BSPG 2019 III.

### Completeness of the taxonomic inventory

We estimated the completeness of Simssee taxonomic inventory based on two complementary methods, assuming teeth/spines represent different individuals. First, rarefaction was used to estimate the impact of sampling effort (specimens) on total taxonomic richness, where a plateau in the curve suggests a saturation trend (Sanders and Hessler 1969; Gotelli and Colwell 2011). Second, we estimated the true (i.e. discoverable) taxonomic richness based on the Chao 1 extrapolation index (Chao and Lee 1992; Colwell and Coddington 1994). The Chao 1 index is a non-parametric method that estimates the taxonomic richness in a given locality based on the number of rare taxa (i.e. with one and two individuals), providing a point estimator and an upper level confidence interval (95%). The completeness of the taxonomic inventory was calculated as the fraction between the observed richness and the extrapolated richness (point estimator and upper level confidence interval). This method has been previously used in paleontological studies to estimate the discoverable species richness (e.g. Rivadeneira and Nielsen 2017).

### Faunal comparison

We compared the faunal composition of Simssee with other early Miocene elasmobranch faunas worldwide by using a comprehensive literature dataset (see references in Table 2). Analyses were carried out at the genus level and pooling nearby localities to reduce identification and sampling biases. Since some localities lack precise age estimations, we only used localities that could be assigned to the Eggenburgian–Ottnangian time span (i.e. lower to middle Burdigalian). We used presence–absence data to estimate differences in generic composition between Simssee and other localities using the Sørensen–Dice similarity index. This index has been widely recommended in the ecological and paleontological literature due to its statistical properties (Hubalek 1982; Murguía and Villaseñor 2003; Hammer and Harper 2006; Jost et al. 2011). However, the Sørensen–Dice index, as any presence–absence similarity index, is sensitive to the completeness of taxonomic inventories (Jost et al. 2011).

### Systematic palaeontology

Class **Chondrichthyes** Huxley, 1880

Subclass **Elasmobranchii** Bonaparte, 1838

Infraclass **Neoselachii** Compagno, 1977

Order **Hexanchiformes** Buen, 1926.

Family **Chlamydoselachidae** Garman, 1884

Genus ***Chlamydoselachus*** Garman, 1884

*Type species. Chlamydoselachus anguineus* Garman, 1884

***Chlamydoselachus ***sp.

Figure 2a, b

**Fig. 2:**
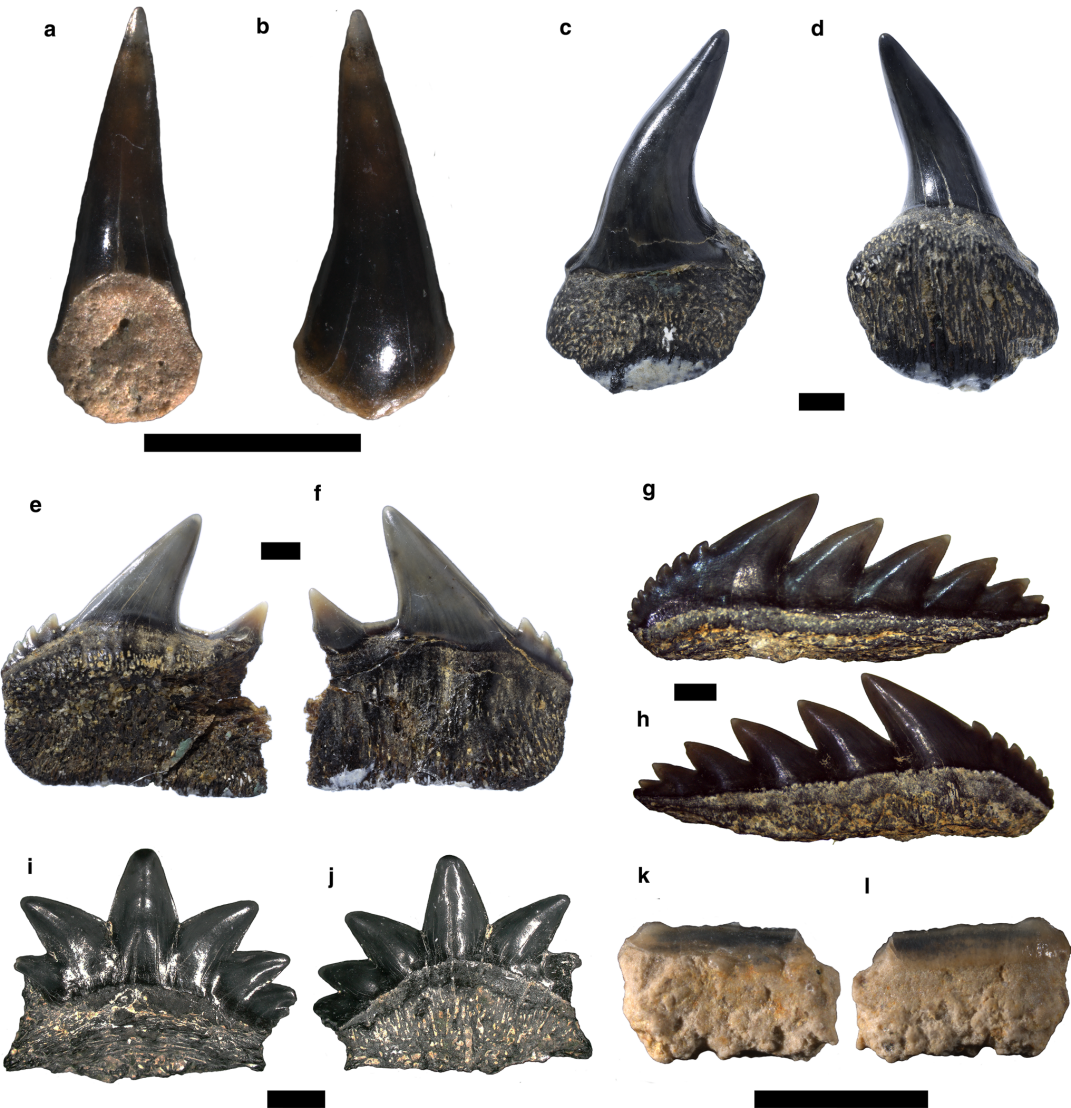
Hexanchiformes. **a**, **b** *Chlamydoselachus* sp., antero-lateral tooth. **c**–**l** *Notorynchus primigenius*: **c**, **d** anterior tooth, **e**, **f** upper antero-lateral tooth, **g**, **h** lower lateral tooth, **i**, **j** lower symphyseal tooth, **k**, **l** commissural tooth. Labial **a**, **c**, **e**, **g**, **i**, **k**; lingual **b**, **d**, **f**, **h**, **j**, **l**. Scale bar 2 mm

*Material.* One antero-lateral tooth—SNSB-BSPG 2019 III-1.

*Description.* The antero-lateral tooth displays a one preserved long and slender cusp, which is lingually bent (Fig. 2a, b). The crown surface is mostly smooth, but faint vertical folds are present at the base of both cusp faces. In profile view, the lingual face is concave, whereas the labial face is convex. Although the root is incomplete and abraded, the small nutritive foramen is still distinguishable.

*Remarks.* The frilled shark *Chlamydoselachus* is currently represented by two species: *C. africana* which occurs in the Southeastern Atlantic, and *C. anguineus*, reported from the western Indian, Eastern Atlantic and Pacific Ocean (Uyeno et al. 1983; Ebert and Compagno 2009). The fossil record of *Chlamydoselachus* extends back to the Upper Cretaceous (Kriwet et al. 2016; Cappetta 2012). Early Miocene records of *Chlamydoselachus* were reported from Austria (Pfeil 1983; Schultz 2013), Germany (Barthelt et al. 1991), and the USA (Phillips et al. 1976).

As the single tooth is abraded and incompletely preserved, it is not possible to identify the specimen at specific level.

Family **Hexanchidae** Gray, 1851

Genus ***Notorynchus*** Ayres, 1855

*Type species. Notorynchus maculatus* Ayres, 1855

***Notorynchus primigenius ***(Agassiz, 1843)

Figure 2c–l

*Material.* Two upper anterior teeth—SNSB-BSPG 2019 III-2, SNSB-BSPG 2019 III-3; 2 upper antero-lateral teeth—SNSB-BSPG 2019 III-4, SNSB-BSPG 2019 III-5; 12 lower antero-lateral teeth—SNSB-BSPG 2019 III-6, SNSB-BSPG 2019 III-7 (4 teeth), SNSB-BSPG 2019 III-8 (7 teeth); 1 commissural tooth—SNSB-BSPG 2019 III-9 and 1 lower symphyseal tooth—SNSB-BSPG 2019 III-10.

*Description.* The upper anterior teeth have a slender and triangular cusp that is distally oriented (Fig. 2c, d). The cutting edges are smooth and do not reach the base of the crown. The root is high and rounded with a convex outline in labial and lingual views.

The upper antero-lateral teeth are labio-lingually compressed, but narrower than the lower teeth (Fig. 2e, f). The crown is composed of a main cusp followed by a secondary cusp that is distally oriented. The root is high and flat with almost straight basal aspect.

The lower antero-lateral teeth are labio-lingually compressed and mesio-distally elongated (Fig. 2g, h). The crown shows a comb-like shape with triangular cusps, which are distally oriented. The most complete specimen bears six cusps decreasing in size distally. The mesial cutting edge is slightly convex with awl-shaped coarse serrations that become finer towards the base of the crown. The root is incomplete, being low and flat with rectilinear basal part.

The symphyseal tooth is mesio-distally compressed with a main straight cusp flanked by two mesial and three distal secondary cusplets (Fig. 2i, j). The mesial secondary cusplets are mesially oriented, whereas the distal ones are distally oriented. The root is low and possibly abraded. The commissural tooth displays a very low crown, mesio-distally elongated (Fig. 2k, l). The distal part of the crown is missing. The root is high and flat.

*Remarks* The genus *Notorynchus* is nowadays only represented by the broadnose sevengill shark *N. cepedianus*, which is a cosmopolitan species occurring in warm temperate to subtropical seas, but which is absent in the North Atlantic and Mediterranean Sea (Compagno et al. 1989; Last and Stevens 2009). having teeth described here similar to *N. primigenius* from the early Miocene of Germany (Reinecke et al. 2011), we determine them as belonging to this species, whose fossil record ranges from the Oligocene to the Miocene (Cappetta 2012). Early Miocene records of *N. primigenius* were also reported from Austria (Schultz 2013), Germany (Probst 1879 as *Notidanus primigenius*; Lutzeier 1922; von Ihering 1927; Barthelt et al. 1991; Scholz and Bienert 1992; Baier et al. 2004; Höltke 2014; Sach 2016), France (Cappetta 1970, 1973, as *Hexanchus primigenius*), Hungary (Kordos and Solt 1984, as *Hexanchus primigenius*; Kocsis 2007), Slovakia (Holec et al. 1995), Switzerland (Leriche 1927; Fischli 1930, as *Notidanus primigenius*; Bolliger et al. 1995) and the USA (Kent 2018).

Order **Squaliformes** Goodrich, 1909

Family **Squalidae** Bonaparte, 1838

Genus ***Squalus*** Linnaeus, 1758

*Type species. Squalus acanthias* Linnaeus, 1758

***Squalus*** sp.

Figure 3a, b

**Fig. 3:**
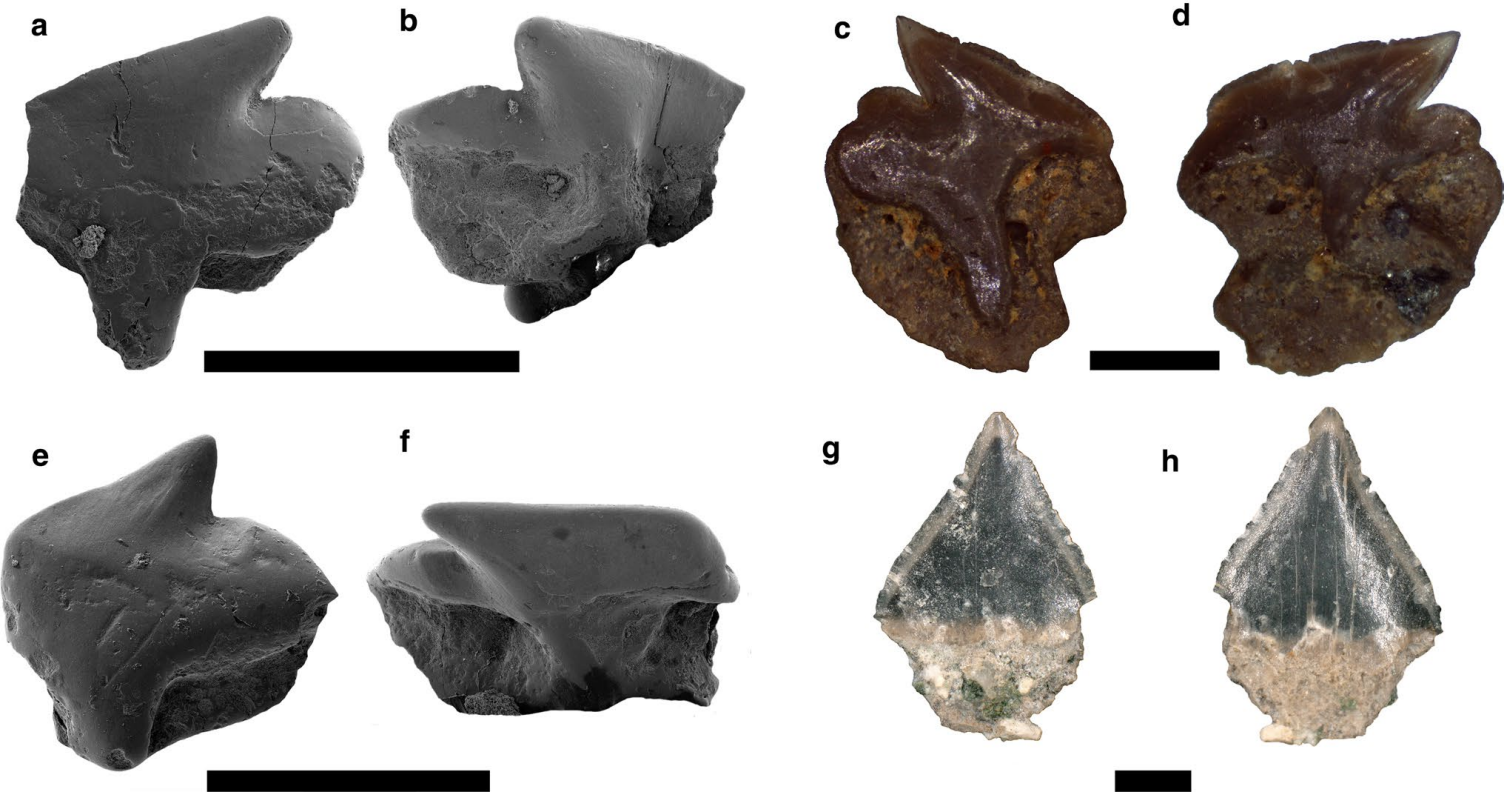
Squaliformes. **a**, **b** *Squalus* sp., antero-lateral tooth, **c**, **d** *Centrophorus granulosus*, lower antero-lateral tooth, **e**, **f** *Deania* sp., lower antero-lateraol tooth, **g**, **h** *Isistius triangulus*, lower anterior tooth. Labial **a**, **c**, **e**, **g**.; lingual **b**, **d**, **f**, **h**. Scale bar 1 mm

*Material.* Two lower antero-lateral teeth—SNSB-BSPG 2019 III-11, SNSB-BSPG 2019 III-12.

*Description.* These teeth show a broad, triangular and strongly distally bent cusp with a rounded apex (Fig. 3a, b). The distal heel is high with a convex outline. The distal and mesial cutting edges are completely smooth. In labial view, a long apron is present. In lingual view, the uvula is abraded but still distinguishable. The root is short.

*Remarks*. The genus *Squalus* is currently represented by 35 species worldwide distributed, including the Mediterranean Sea (Pollerspöck and Straube 2019). *Squalus* species inhabit the upper continental and insular slopes at tropical to temperate latitudes. The fossil record of *Squalus* extends back to the Upper Cretaceous. Early Miocene records were reported from Austria (Schultz 2013), Chile (Suarez et al. 2006; Villafaña et al. 2019), France (Ledoux 1972), Hungary (Kocsis 2007), Germany (Probst 1879; von Ihering 1927; Barthelt et al. 1991; Reinecke et al. 2008; Pollerspöck and Beaury 2014; Pollerspöck and Straube 2017), India (Mondal et al. 2009), Slovakia (Holec et al. 1995), Switzerland (Bolliger et al. 1995) and the USA (Emry and Eshelman 1998; Purdy et al. 2001; Kent 2018).

The teeth reported here differ from those previously described from Germany (Reinecke et al. 2011; Pollerspöck and Straube 2017). In those specimens, the apex of the crown is very acute and the apron is narrower. However, differences in the material described here could be related also to a taphonomic effect. Therefore, as the teeth are very abraded and lack diagnostic characters, our specimens can be only identified at the genus level.

Family **Centrophoridae** Bleeker, 1859

Genus ***Centrophorus*** Müller and Henle, 1837

*Type species. Squalus granulosus* Bloch and Schneider, 1801

***Centrophorus granulosus*** (Bloch and Schneider, 1801)

Figure 3c, d

*Material.* Eleven lower antero-lateral teeth—SNSB-BSPG 2019 III-13, SNSB-BSPG 2019 III-14 (2 teeth), SNSB-BSPG 2019 III-15 (8 teeth); and 3 upper lateral teeth—SNSB-BSPG 2019 III-16, SNSB-BSPG 2019 III-17 (2 teeth).

*Description.* The lower antero-lateral teeth are labio-lingually compressed with a broad and distally inclined cusp (Fig. 3c, d). The mesial edge is faintly sigmoidal and serrated, whereas the distal edge is slightly convex and smooth. The distal heel is notched and convex without any serrations. The apron is long and broader at its base with a rounded end. On the lingual face, a short uvula with a deep infundibulum is present just below its lower extremity. The distal part of the root is larger than the mesial one and displays some foramina. In labial view, the mesial part of the root displays a large foramen and has a concave contour. In upper lateral teeth, the crown is higher than broad. The mesial edge is slightly sigmoidal and serrated. The distal heel is short and strongly convex with weak serrations. The root is very abraded and covered by sediment. Apron, uvula and root foramina are not distinguishable.

*Remarks.* The genus *Centrophorus* is currently represented by 13 species with global distributions (Pollerspöck and Straube 2019). These mid- to deep-water sharks inhabit tropical to temperate environments (Compagno et al. 2005; White et al. 2013). The fossil record of *Centrophorus* extends back into the upper Cretaceous (Cappetta 2012). Early Miocene records of *Centrophorus* were reported from Austria (Pfeil 1983; Schultz 2013), Colombia (Carrillo-Briceño et al. 2016a), Germany (Probst 1879; Fischli 1930; Barthelt et al. 1991; Scholz and Bienert 1992; Baier et al. 2004; Pollerspöck and Beaury 2014; Sach 2016; Pollerspöck and Straube 2017), Switzerland (Bolliger et al. 1995; Jost et al. 2016), Slovakia (Holec et al. 1995 as *Squalus* sp.) and the USA (Phillips et al. 1976).

According to Vialle et al. (2011), the serrated mesial cutting edge and the absence of folds on the uvula are the diagnostic characters that separate *C. granulosus* from other species. Thereby, these characters that are present in our material allow us to identify it at species level.

Genus ***Deania*** Jordan and Snyder, 1902

*Type species. Deania eglantina* Jordan and Snyder, 1902

***Deania*** sp.

Figure 3e, f

*Material.* One lower tooth—SNSB-BSPG 2019 III-18.

*Description.* The lower tooth is labio-lingually compressed with a short, narrow and distally oriented cusp (Fig. 3e, f). The cutting edges are completely smooth. The mesial cutting edge is convex in its lower part and slightly straight in its upper part. The distal cutting edge is slightly convex. The distal heel is convex and serrated. The apron is short and broad, reaching the base of the root. In lingual view, the uvula is short. The root is short, abraded and covered with sediment; thus, the infundibulum and foramina are not distinguished.

*Remarks.* The genus *Deania* is currently represented by four species (*D. calcea*, *D. hystricosa*, *D. profundorum* and *D. quadrispinosa*) occurring in the Atlantic, Indian, and Pacific oceans, but not in the Mediterranean Sea (Compagno 1984a; Akhilesh et al. 2010). The fossil record of *Deania* extends back into the lower Paleocene (Cappetta 2012). Early Miocene records were only reported from Austria (Pfeil 1983; Schultz 2013; Pollerspöck et al. 2018), Germany (Pollerspöck and Straube 2017) and Switzerland (Bolliger et al. 1995). However, according to Reinecke et al. (2011), *Deania* also occurrs in different localities of the early Miocene in the Paratethys and Mediterranean seas.

Considering the narrow cusp and the smooth mesial cutting edge, the tooth described here can be unambiguously identified as belonging to the genus *Deania*. However, due to the lack of diagnostic characters and the small number of specimens available for this study, it is not possible to identify it at specific level.

Family **Dalatiidae** Gray, 1851

Genus ***Isistius*** Gill, 1864

*Type species*. *Scymnus brasiliensis* Quoy and Gaimard, 1824

***Isistius triangulus*** (Probst, 1879)

Figure 3g, h

*Material.* Two lower antero-lateral teeth—SNSB-BSPG 2019 III-19, SNSB-BSPG 2019 III-20.

*Description.* The crowns of the two antero-lateral teeth are abraded and the root is incomplete (Fig. 3 g, h). The crown is labio-lingually compressed and triangular. The cutting edges are smooth and almost straight. The crown slightly overhangs the root distally and mesially. The root is high, flat and covered with sediment; thus, the median foramina cannot be distinguished.

*Remarks.* The genus *Isistius* is currently represented by three species: *I. brasiliensis*, *I. labialis* and *I. plutodus* (de Figueiredo and de Carvalho 2018)*.* The cookie cutter shark (*I. brasiliensis*) has a wide geographic distribution in tropical and subtropical environments, whereas the largetooth cookie cutter shark (*I. plutodus*) has been reported from the Atlantic and Northwest Pacific oceans (Compagno 1984a; Compagno et al. 2005). The fossil record of *I. triangulus* ranges from the early Miocene to the early Pliocene (Cappetta 2012). Early Miocene records are from Austria (Schultz 2013), France (Cappetta 1970), Germany (Probst 1879, as *Scymnus triangulus*; von Ihering 1927; Barthelt et al. 1991; Pollerspöck and Beaury 2014; Sach 2016; Pollerspöck and Straube 2017), Hungary (Kocsis 2007), Portugal (Antunes and Jonet 1970), Slovakia (Holec et al. 1995) and Switzerland (Leriche 1927; Jost et al. 2016).

According to Laurito (1997, 1999), the fossil species *I. triangulus* can be distinguished from the extant *I. brasiliensis* and *I. plutodus* by its different crown shape. In *I. triangulus*, the crown edges form an equilateral triangle (Carrillo-Briceño et al. 2014; Pérez and Marks 2017), whereas it is isosceles in *I. brasiliensis* and *I. plutodus*.

Order **Squatiniformes** Buen, 1926

Family **Squatinidae** Bonaparte, 1838

Genus ***Squatina*** Dumeril, 1806

*Type species. Squalus squatina* Linnaeus, 1758

***Squatina ***sp*.*

Figure 4a, b

**Fig. 4:**
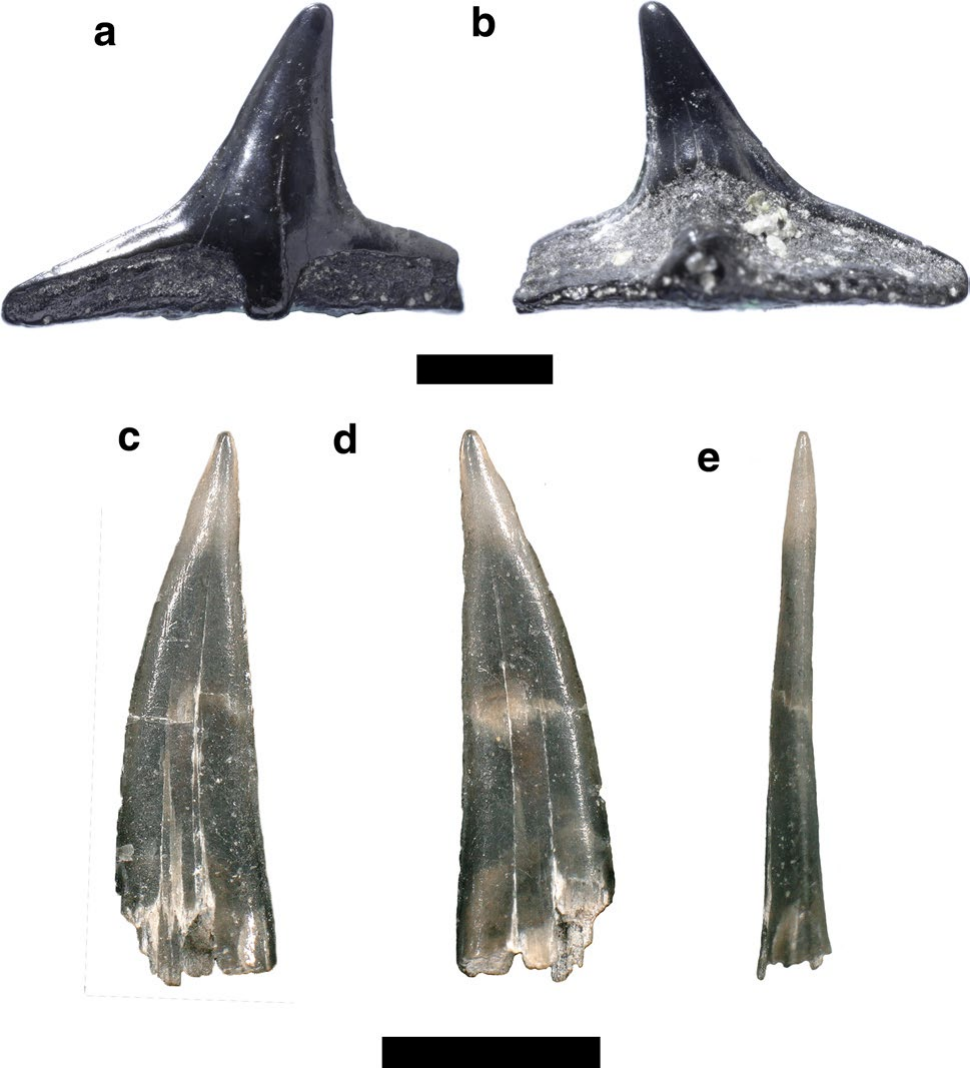
Squatiniformes. **a**, **b** *Squatina* sp., lateral tooth, Pristiophoriformes. **c**–**e** *Pristiophorus* sp., rostral spine. Labial: **a**; lingual: **b**; dorsal: **c**; ventral: **d**; anterior: **e**. Scale bar 2 mm

*Material.* Four lateral teeth—SNSB-BSPG 2019 III-21, SNSB-BSPG 2019 III-22 (two teeth), SNSB-BSPG 2019 III-23.

*Description.* The lateral teeth are broader than high (Fig. 4a, b). The crown is rather low, slender and distally inclined. The cutting edges are smooth. The mesial and distal heels are elongated and low with sharp cutting edges. In labial view, the apron is short and basally rounded. The mesial and distal crown–root boundaries are slightly concave. The lingual protuberance is massive with a well-developed foramen at its extremity. In profile view, the lingual crown face is slightly concave, whereas the labial crown face is convex. The root is high and broad in lingual view. In labial view, the root face is slightly straight basally.

*Remarks.* The genus *Squatina* is currently represented by 24 species with global distributions in temperate and tropical seas (Pollerspöck and Straube, 2019). In the Mediterranean Sea, *Squatina* is represented by the angel shark (*S. squatina*), the sawback angelshark (*S. aculeata*) (Serena 2005; Ebert and Stehmann 2013) and the smoothback angelshark (*S. oculata*) (Ergüden et al. 2019). The genus extends back to the Early Cretaceous (Klug and Kriwet 2013). Early Miocene records of *Squatina* were reported from Austria (Brzobohatý and Schultz 1971; Schultz 2013), Chile (Suarez et al. 2006; Villafaña et al. 2019), France (Cappetta 1970, 1973; Canevet 2011), Germany (Probst 1879; Lutzeier 1922; von Ihering 1927; Barthelt et al. 1991; Scholz and Bienert 1992; Baier et al. 2004; Reinecke et al. 2011; Pollerspöck and Beaury 2014; Sach 2016), Hungary (Kordos and Solt 1984; Kocsis 2007), Peru (Landini et al. 2019), Portugal (Antunes and Jonet 1970), Switzerland (Leriche 1927; Fischli 1930; Bolliger et al. 1995), Slovakia (Holec et al. 1995) and the USA (Phillips et al. 1976; Case 1980).

Order **Pristiophoriformes** Berg, 1958

Family **Pristiophoridae** Bleeker, 1859

Genus ***Pristiophorus*** Müller and Henle, 1837

*Type species. Pristis cirratus* Latham, 1794

***Pristiophorus ***sp.

Figure 4c–e

*Material.* Two rostral spines—SNSB-BSPG 2019 III-24, SNSB-BSPG 2019 III-25.

*Description.* The rostral spines are abraded and the basal peduncle is missing (Fig. 4c–e). The enameloid cap is long, slender and dorso-ventrally flattened with smooth cutting edges. The enameloid is smooth and devoid of any ornamentation. The spine is slightly bent towards the rear.

*Remarks.* The genus *Pristiophorus* is currently represented by seven species that are distributed in temperate and subtropical regions (Compagno 2001). This genus is present in the western Pacific, western Central Atlantic, and Indian oceans, but not in the Mediterranean Sea (Compagno 1998; Last and Stevens 2009; Yearsley et al. 2008). The fossil record of *Pristiophorus* extends back to the lower Cretaceous (Cappetta 2012). Early Miocene records were reported from Australia (Fitzgerald 2004), Austria (Schultz 2013), Chile (Suarez et al. 2006; Villafaña et al. 2019), Colombia (Carrillo-Briceño et al. 2016a), Germany (von Ihering 1927; Barthelt et al. 1991; Reinecke et al. 2011; Pollerspöck and Beaury 2014; Sach 2016; Pollerspöck and Straube 2017), Slovakia (Underwood and Schlögl 2013) and Switzerland (Fischli 1930; Jost et al. 2016).

The rostral spine reported here can be unambiguously identified as belonging to the genus *Pristiophorus*. According to Underwood and Schlögl (2013) and Engelbrecht et al. (2017), *Pristiophorus* species erected on the sole basis of rostral teeth should be considered as nomina dubia because of the lack of specific diagnostic characters. Thereby, the oral teeth should be used as comparative material instead of rostral spines. For this reason, identification at specific level of the single *Pristiophorus* spine reported here is not possible.

Order **Lamniformes** Berg, 1937

Family **Mitsukurinidae** Jordan, 1898

Genus ***Mitsukurina*** Jordan, 1898

*Type species. Mitsukurina owstoni* Jordan, 1898

***Mitsukurina lineata*** (Probst, 1879)

Figure 5a, b

**Fig. 5:**
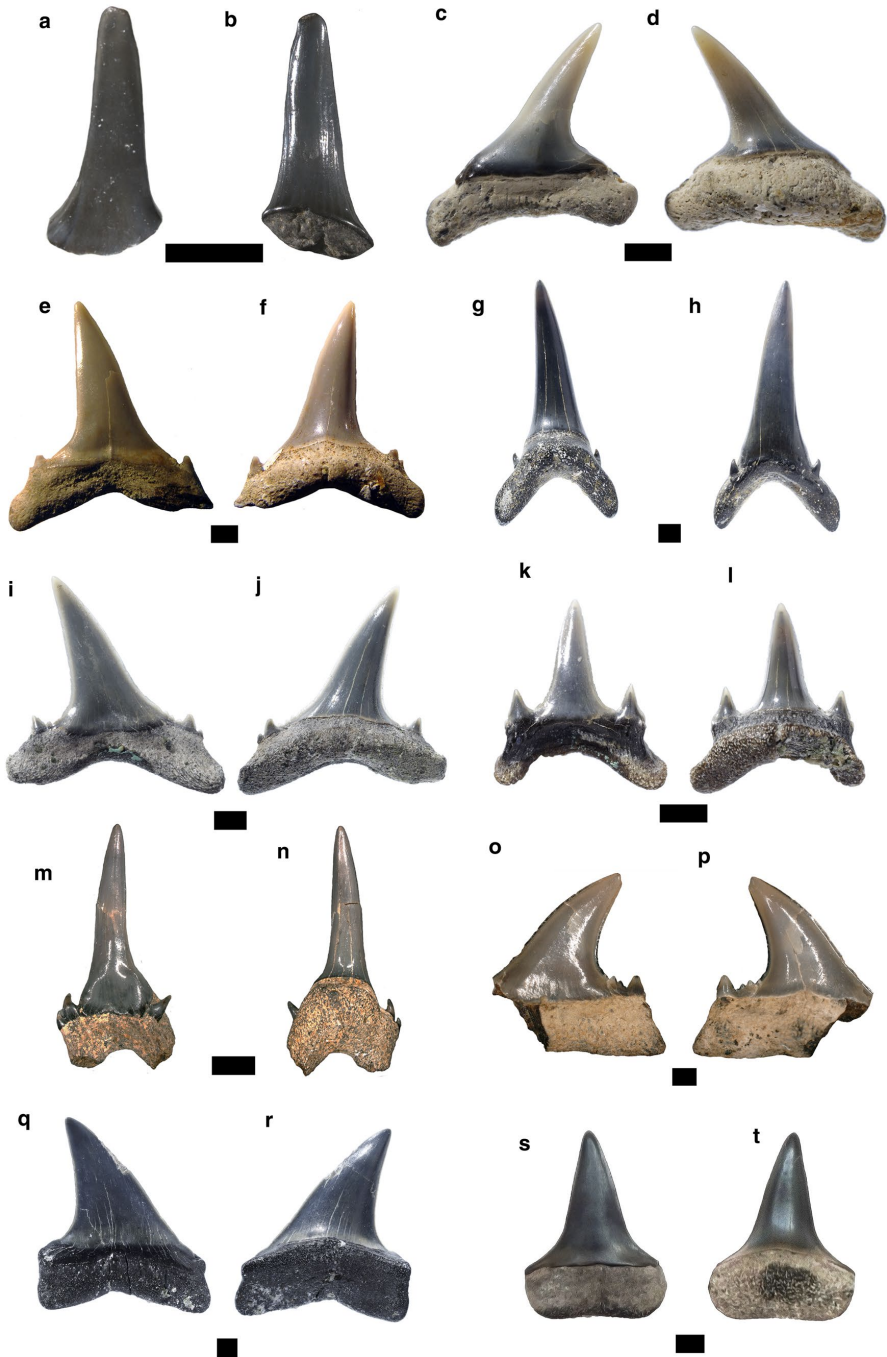
Lamniformes. **a**–**d** *Mitsukurina lineata*, **a**, **b** upper lateral tooth, *Alopias exigua*, **c**, **d** lower lateral teeth, **e**, **f** *Araloselachus cuspidatus*, **g**, **l** *Carcharias acutissima*, **g**, **h** upper lateral tooth, **i**, **j** lower anterior tooth, **k**, **l** lower latero-posterior tooth, **m**–**p** *Odontaspis molassica*, **q**–**t** *Carcharodon hastalis*, **q**, **r** upper lateral, lower. Labial: **a**, **c**, **e**, **g**, **i**, **l**, **n**; lingual: **b**, **d**, **f**, **h**, **j**, **k**, **m**. Scale bar 2 mm

*Material.* Two upper anterior teeth—SNSB-BSPG 2019 III-26, SNSB-BSPG 2019 III-27.

*Description.* The teeth have a high, slender and in profile view slightly sigmoidal cusp with a broad base (Fig. 5a, b). In labial view, the crown surface is almost completely smooth with only few short folds at its base. In lingual view, strong longitudinal folds extend from its base to the middle part of the cusp. Both faces of the crown are slightly convex transversely. The root is missing.

*Remarks.* The genus *Mitsukurina* is represented today only by the goblin shark *M. owstoni*, occurring in the Atlantic, Pacific and western Indian oceans, but absent in the south-eastern Pacific and Mediterranean Sea (Last and Stevens 1994; Compagno 2001). The fossil record of *Mitsukurina lineata* dates back to the early Miocene of Europe (Cappetta 2012).

Early Miocene records of this species are from Austria (Schultz 2013), France (Joleaud 1912; Cappetta 1975), Germany (Probst 1879, as *Lamna lineata*; Lutzeier 1922; Barthelt et al. 1991; Scholz and Bienert 1992; Baier et al. 2004; Höltke 2014; Pollerspöck and Beaury 2014; Sach 2016), Hungary (Kocsis 2007), Slovakia (Holec et al. 1995) and Switzerland (Bolliger et al. 1995; Jost et al. 2016).

Family **Alopiidae** Bonaparte, 1838

Genus ***Alopias*** Rafinesque, 1810

*Type species. Alopias macrourus* Rafinesque, 1810

***Alopias exigua*** (Probst, 1879)

Figure 5c, d

*Material.* Five lower lateral teeth—SNSB-BSPG 2019 III-28, SNSB-BSPG 2019 III-29.

*Description.* The teeth have a slender, acute and distally inclined cusp with a broad base (Fig. 5c, d). The mesial cutting edge is rather straight, whereas the distal one is strongly concave. Both cutting edges are sharp and reach the base of the crown. The labial crown face is almost flat, whereas the lingual face is convex. Both faces are devoid of any ornamentation. In labial view, the crown/root boundary is straight. A narrow and rather straight crown neck separates the labial crown face from the root. The root lobes are short and well divergent with rounded extremities.

*Remarks.* The genus *Alopias* is currently represented by three species: *A. pelagicus*, *A. superciliosus*, and *A. vulpinus.* The pelagic thresher (*A. pelagicus*) is distributed in the Indo-Pacific and eastern Pacific. The bigeye thresher (*A. superciliosus*) and the thresher (*A. vulpinus*) have a wide distribution in tropical and temperate oceans, including the Mediterranean Sea (Last and Stevens 1994; Compagno et al. 2005). The fossil record of *A. exigua* ranges from the early Oligocene to the middle Miocene (Cappetta 2012). Early Miocene records of *A. exigua* were reported from Austria (Brzobohatý and Schultz 1971), France (Cappetta 1970), Germany (von Ihering 1927; Barthelt et al. 1991; Baier et al. 2004; Reinecke et al. 2011; Höltke 2014; Pollerspöck and Beaury 2014; Sach 2016) and Hungary (Kordos and Solt 1984; Kocsis 2007).

The extinct *A. exigua* can be distinguished from the extant species by its narrower cusp and lower root. The teeth described here match perfectly with those reported from the early Miocene of northern Germany by Reinecke et al. (2011).

Family **Odontaspididae** Müller and Henle, 1838

Genus ***Araloselachus*** Glikman, 1964

*Type species. Araloselachus agespensis* Glikman, 1964

***Araloselachus cuspidatus*** (Agassiz, 1843)

Figure 5e, f

*Material.* Six upper antero-lateral teeth—SNSB-BSPG 2019 III-30, SNSB-BSPG 2019 III-31, SNSB-BSPG 2019 III-32 (four teeth); and one anterior tooth—SNSB-BSPG 2019 III-33.

*Description.* The upper antero-lateral teeth have a triangular and distally inclined cusp (Fig. 5e, f). The mesial cutting edge is slightly concave at its base and convex at its upper part. In profile view, the crown is rather straight. The distal cutting edge is concave at its base and straight in its medial and upper parts. A pair of low lateral cusplets are present, which are triangular and devoid of any ornamentation. The root is low with short and well-separated lobes.

The anterior tooth (not figured) is very abraded and part of the apex is missing. The crown is triangular, high and robust. In profile view, the cusp also is straight. The labial crown/root boundary is concave and the labial face overhangs the root in its medial part. The enameloid surface is completely smooth on both crown faces. The lateral cusplets are broken. The root is high with well-separated and long lobes.

*Remarks. A. cuspidatus *is common in Oligocene and Miocene fossiliferous sites (Cappetta 2012). Early Miocene records are from Australia (Pledge 1967), Austria (Brzobohatý and Schultz 1971), France (Cappetta 1970, 1973; Goedert et al. 2017), Germany (Barthelt et al. 1991, as *Synodontaspis cuspidata*; Sach and Heizmann 2001; Sach 2016, as *Carcharias cuspidatus*), Hungary (Kocsis 2007, as *Carcharias cuspidatus*), Italy (Marsili et al. 2007), Portugal (Zbyszewski 1949), Slovakia (Holec et al. 1995), Switzerland (Bolliger et al. 1995, as *Carcharias cuspidatus*) and the USA (Case 1980; Kent 2018).

According to Cappetta (2012), the genus *Araloselachus* has sufficient morphological characters to be separated from other odontaspid genera. In *A. cuspidatus*, features of the anterior teeth are used to separate the species from other odontaspids. In this species, the teeth are more robust and stronger, the crown is straight in profile view and the enameloid surface is completely smooth. The teeth reported herein are similar to those described from the middle Miocene of Hungary (Szabó and Kocsis 2016).

Genus ***Carcharias*** Rafinesque, 1810

*Type species. Carcharias taurus* Rafinesque, 1810

***Carcharias acutissimus*** (Agassiz, 1843)

Figure 5g–l

*Material.* 44 anterior teeth—SNSB-BSPG 2019 III-34, SNSB-BSPG 2019 III-35 (4 teeth), SNSB-BSPG 2019 III-36 (39 teeth); 31 upper lateral teeth—SNSB-BSPG 2019 III-37, SNSB-BSPG 2019 III-38 (5 teeth), SNSB-BSPG 2019 III-39 (25 teeth); and 20 lower lateral teeth—SNSB-BSPG 2019 III-40, SNSB-BSPG 2019 III-41 (5 teeth), SNSB-BSPG 2019 III-42 (14 teeth).

*Description.* The anterior teeth show an elongated and slender cusp with a strong sigmoidal profile (Fig. 5g, h). The cutting edges are smooth and do not reach the base of the crown. The crown/root boundary is strongly concave in labial view. One to two pairs of sharp lateral cusplets are present, which are lingually bent. The root is high with two long and well-separated lobes. The lingual protuberance displays a well-developed nutritive groove.

In the upper lateral teeth, the crown is triangular and distally inclined (Fig. 5i, j). In profile view, the crown is straight. In lingual view, the enameloid surface shows weakly vertical folds at its base. The crown/root boundary is slightly concave in labial view.

The lower lateral teeth show a straight, rather low and triangular cusp (Fig. 5k, l). There are one to three pairs of lateral cusplets, which are high and triangular in labial view. The crown/root boundary is strongly concave. The root is low with two short and well-separated lobes. The lingual protuberance is bifurcated with a nutritive groove.

*Remarks.* The fossil record of *C. acutissimus* ranges from the Oligocene to the Pliocene (Cappetta 2012). Early Miocene records were reported from Austria (Schultz 2013), Costa Rica (Laurito et al. 2014), France (Chevalier 1961; Cappetta 1970, as *Odontaspis acutissima*), Germany (Barthelt et al. 1991, as *Synodontaspis acutissima*; Baier et al. 2004; Höltke 2014), Hungary (Kordos and Solt 1984, as *Odontaspis acutissima*; Kocsis 2007), Italy (Marsili et al. 2007), Slovakia (Holec et al. 1995, as *Synodontaspis acutissima*), Switzerland (Leriche 1927; Fischli 1930) and the USA (Case 1980, as *Odontaspis acutissima*). Although teeth of *C. taurus* share some similarities with those of *C. acutissimus* (Arambourg 1952), no detailed studies about their morphological characters have been carried out so far. The teeth described here are similar to those from the early Miocene of Italy (Marsili et al. 2007) and the middle Miocene of Hungary (Szabó and Kocsis 2016).

Genus ***Odontaspis*** Agassiz, 1843

*Type species. Squalus ferox* Risso, 1810

***Odontaspis molassica*** (Probst, 1879)

Figure 5m–p

*Material.* Two anterior teeth—SNSB-BSPG 2019 III-43, SNSB-BSPG 2019 III-44; and two upper antero-lateral teeth—SNSB-BSPG 2019 III-45, SNSB-BSPG 2019 III-46.

*Description.* The anterior teeth have a high and slender cusp with a slightly sigmoidal profile (Fig. 5m, n). The enameloid surface is completely smooth on both cusp faces. The two pairs of lateral cusplets are high and sharp. The cutting edges are smooth and do not reach the base of the crown. The lingual protuberance is very strong and it is divided by a nutritive groove. The crown/root boundary is slightly concave. The root is high and massive in lingual view. Both lobes are well separated, but lack their extremities.

The upper antero-lateral tooth has a triangular cusp, which is strongly distally oriented (Fig. 5o, p). Three pairs of lateral cusplets are present distally. The mesial cutting edge is convex, whereas the distal one is concave. The crown/root boundary is straight in both faces. The root is rather low and straight. The mesial part of the crown and root is broken.

*Remarks. Odontaspis molassica *is not very common in the fossil record. Early Miocene finds are from France (Cappetta 1970, 1973), Germany (Probst 1879; Barthelt et al. 1991; Baier et al. 2004, Sach 2016) and Portugal (Antunes et al. 1981).

According to Reinecke et al. (2011), the anterior teeth of *O. molassica* can be distinguished from *Carcharias* species (e.g. *C. gustrowensis*) by their more slender and higher cusp. Additionally, the crown surface in *O. molassica* is always smooth on both cusps faces in lateral and anterior teeth. Finally, the teeth described here have a slightly concave to straight crown/root boundary in labial view. All these characters were originally highlighted by Probst (1879) and later confirmed by Bracher and Unger (2007).

Family **Lamnidae** Müller and Henle, 1838

Genus ***Carcharodon*** Müller and Henle, 1838

*Type species. Squalus carcharias* Linnaeus, 1758

***Carcharodon hastalis*** (Agassiz, 1838)

Figure 5q–t

*Material.* One upper lateral—SNSB-BSPG 2019 III-47; and one lower lateral tooth—SNSB-BSPG 2019 III-48.

*Description.* The upper lateral tooth displays a triangular cusp with smooth cutting edges (Fig. 5q, r). The mesial cutting edge is slightly convex, whereas the distal one is concave. The enameloid surface is completely smooth and the labial face is flat. The root is high with short lobes.

In the lower lateral tooth, the cusp is also triangular, but straight and narrower than the upper tooth (Fig. 5s, t). The basal part of the mesial and distal edges is concave. The root is high and flat with a straight basal part, probably eroded.

*Remarks.* The taxonomic classification of *C. hastalis* has been widely debated in the last years (see Purdy et al. 2001; Cappetta 2012; Cione et al. 2012; Ehret et al. 2012; Boessenecker et al. 2019). Ehret et al (2012) proposed a reconstruction of the evolutionary history of the genus *Carcharodon* based on dental characters shared between the fossil and extant species. According to these authors, the non-serrated C. *hastalis* evolved into the semi-serrated C. *hubbelli* and then to the fully serrated *C.carcharias.* Therefore, the evolutionary transition from *C. hastalis* to *C. carcharias* occurred within a span of 6.9–5.3 Ma. (Long et al. 2014; Boessenecker et al. 2019).

The fossil record of *C. hastalis* ranges from the Miocene to the Pliocene (Cappetta 2012). Early Miocene records were reported from Argentina (Scasso and Castro 1999), Austria (Schultz 2013), Chile (Suarez et al. 2006), Egypt (Cook et al. 2010), France (Cappetta 1970), Germany (Barthelt et al. 1991, as *Isurus hastalis*; Baier et al. 2004; Reinecke et al. 2011); Sach 2016), Hungary (Kordos and Solt 1984, as *Isurus hastalis*; Kocsis 2007), Italy (Marsili et al. 2007), Peru (Bianucci et al. 2018; Landini et al. 2019, as *Cosmopolitodus hastalis*), Slovakia (Koch 1904, as *Isurus hastalis*), Spain (Vicens and Rodríguez-Perea 2003, as *Isurus hastalis*), Switzerland (Leriche 1927) and the USA (Purdy 1998; Purdy et al. 2001; Kent 2018).

Order **Carcharhiniformes** Compagno, 1973

Family **Carcharhinidae** Jordan and Evermann, 1896

Genus ***Carcharhinus*** de Blainville, 1816

*Type species. Carcharias melanopterus* Quoy and Gaimard, 1824

***Carcharhinus priscus*** (Agassiz, 1843)

Figure 6a, d

**Fig. 6:**
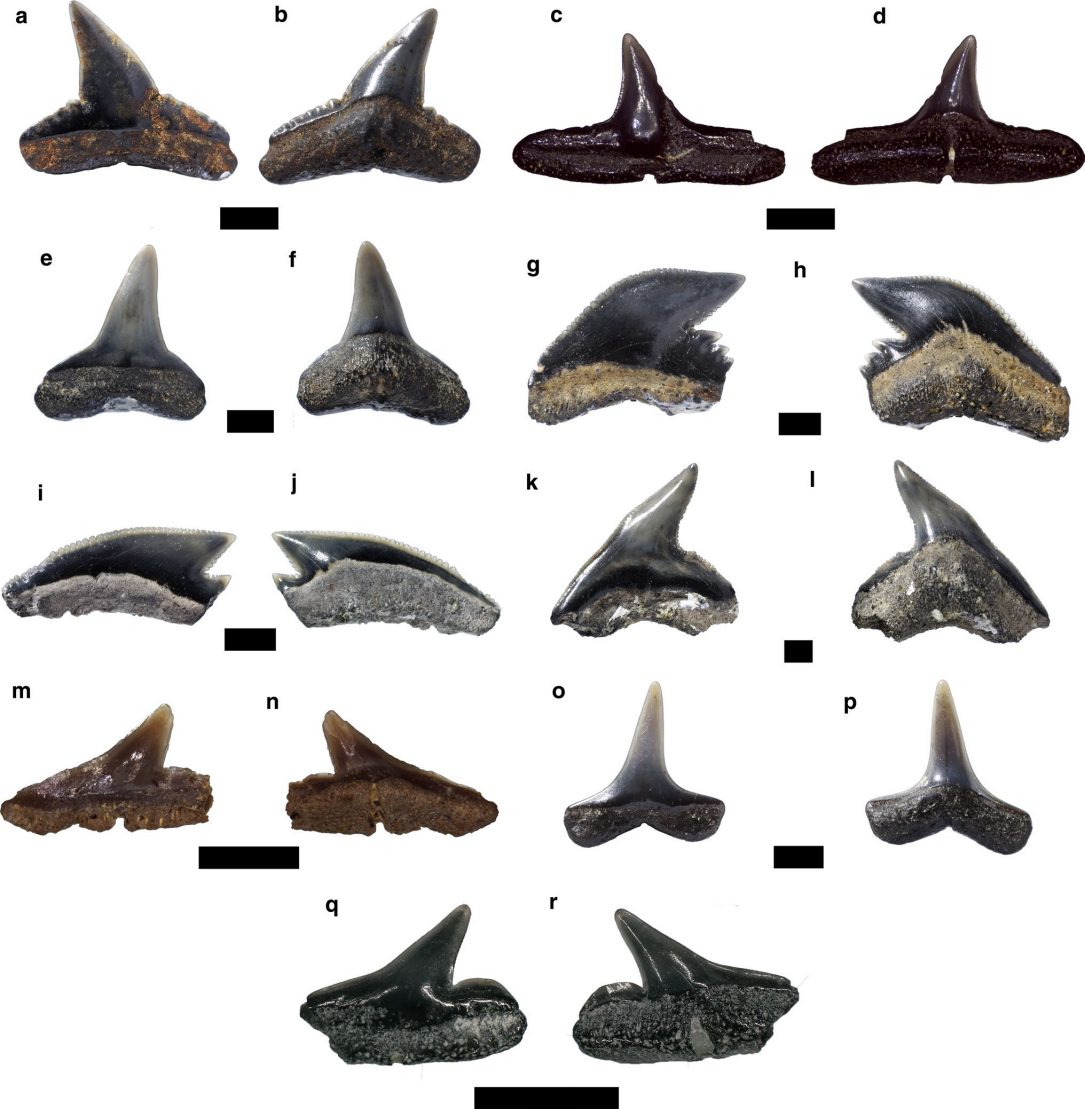
Carcharhiniformes. **a**–**d** *Carcharhinus priscus*, **a**, **b** upper antero-lateral tooth, **c**, **d** lower antero-lateral tooth, **e**, **f** *Carcharhinus* sp., **g**–**j** *Galeocerdo aduncus*, **k**, **l** *Physogaleus contortus*, **m**, **n** *Rhizoprionodon fischeri*, **o**, **p** *Isogomphodon acuaris*, **q**, **r** *Sphyrna* sp. Labial: **a**, **c**, **e**, **g**, **i**, **k**, **m**, **o**; lingual: **b**, **d**, **f**, **h**, **j**, **l**, **n**, **p**. Scale bar 2 mm

*Material.* 25 upper antero-lateral teeth—SNSB-BSPG 2019 III-49, SNSB-BSPG 2019 III-50 (6 teeth), SNSB-BSPG 2019 III-51 (18 teeth); and 14 lower teeth—SNSB-BSPG 2019 III-52, SNSB-BSPG 2019 III-53 (13 teeth).

*Description.* The upper antero-lateral teeth have a broad and triangular cusp, which is distally inclined (Fig. 6a, b). The cutting edges are continuously serrated along the main cusp and on the heels. The root is high in lingual view with well–separated root lobes. A shallow nutritive groove is present in the lingual root protuberance.

In the lower teeth, the cusp is narrower and also distally inclined with broad lateral heels (Fig. 6c, d). The cutting edges are faintly serrated in the main cusp and heels. In lingual view, the root is mesio-distally extended with a distinct furrow and foramen. The basal face of the root is straight basally.

*Remarks. Carcharhinus priscus *is one the most common species reported from the Neogene of the Mediterranean Sea and Paratethys (Cappetta 2012; Szabó and Kocsis 2016), but might represent a wastebasket taxon. This fossil species range from the early Miocene to Pliocene according to Reinecke et al. (2011). Early Miocene records were reported from Austria (Schultz 2013), Brazil (Toledo 1989), France (Cappetta 1970, 1973), Germany (Barthelt et al. 1991; Scholz and Bienert 1992; Baier et al. 2004; Reinecke et al. 2011; Pollerspöck and Beaury 2014; Sach 2016), Hungary (Kocsis 2007), Pakistan (Welcomme et al. 1997), Portugal (Antunes et al. 1981), Saudi Arabia (Thomas 1982), Slovakia (Holec et al. 1995), Spain (Vicens and Rodríguez-Perea 2003), Switzerland (Bolliger et al. 1995) and the USA (Case 1980; Kent 2018).

According to Maisch et al. (2018), the serrated cutting edges and the absence of a notch separating the main cusp and the tooth shoulders allow to separate *C. priscus* from other species of *Carcharhinus*. The teeth reported herein share similarities with those from the early Miocene of Northern Germany (see Reinecke et al. 2011) and are therefore assigned to this species.

***Carcharhinus*** sp.

Figure 6e, f

*Material.* 14 upper antero-lateral teeth—SNSB-BSPG 2019 III-54, SNSB-BSPG 2019 III-55 (13 teeth); and three lower antero-lateral teeth—SNSB-BSPG 2019 III-56.

*Description.* The upper antero-lateral teeth display a triangular and distally inclined cusp (Fig. 6e, f). The cutting edges are completely smooth, probably eroded. The heels are low and slightly convex in labial view. The root is high in lingual view with well-separated lobes. In the lower antero-lateral teeth, the crown is rather low and upright. The cutting edges are also smooth along the cusp and crown shoulders. The root is low with well-separated lobes.

*Remarks.* The genus *Carcharhinus* is currently represented by 35 species with global distributions (Pollerspöck and Straube 2019). In the Mediterranean Sea, the sandbar shark (*C. plumbeus*), the bignose shark (*C. altimus*), the copper shark (*C. brachyurus*), the silky shark (*C. falciformis*), the spinner shark (*C. brevipinna*), the blacktip shark (*C. limbatus*), blacktip reef shark (*C. melanopterus*) and the dusky shark (*C. obscurus*) have been reported up to now (Garibaldi and Relini 2012; Froese and Pauly 2019). The fossil record of *Carcharhinus* extends back to the middle Eocene (Kriwet 2005; Cappetta 2012; Underwood and Gunter 2012). Early Miocene records of *Carcharhinus* were widely reported from Europe and America (Cappetta 1970, 2012; Case 1980; Barthelt et al. 1991; Suarez et al. 2006; Reinecke et al. 2011; Schultz 2013).

Genus ***Galeocerdo*** Müller and Henle, 1837

*Type species. Squalus arcticus* Faber, 1829

***Galeocerdo aduncus*** Agassiz, 1843

Figure 6g–j

*Material.* Eight antero-lateral teeth—SNSB-BSPG 2019 III-57, SNSB-BSPG 2019 III-58, SNSB-BSPG 2019 III-59 (six teeth); and one posterior tooth—SNSB-BSPG 2019 III-60.

*Description.* The antero-lateral teeth have a triangular and broad cusp, which is strongly distally inclined (Fig. 6g, h). The mesial cutting edge is long and sigmoidal, whereas the distal one is short and slightly convex. Both cutting edges are strongly serrated from the base to the middle of the cusp, being only faintly serrated in the apex. The distal heel is high and strongly serrated. The root is very high in lingual view and low in labial face.

The posterior tooth is broader than high (Fig. 6i, j). The crown is low, triangular and strongly distally bent. The mesial cutting edge is convex, whereas the distal one is straight. Both cutting edges are serrated. The distal heel is short, low and also serrated. The root is higher than the crown in lingual view.

*Remarks. Galeocerdo aduncus* is common in Miocene deposits (Cappetta 2012). Early Miocene records were reported from Austria (Schultz 2013; Pollerspöck et al. 2018), France (Cappetta 1970), Germany (Probst 1879; Lutzeier 1922; Barthelt et al. 1991; Scholz and Bienert 1992; Baier et al. 2004; Reinecke et al. 2011; Sach 2016), Hungary (Kordos and Solt 1984; Kocsis 2007), Italy (Marsili et al. 2007), Malta (Ward and Bonavia 2001), Portugal (Antunes et al. 1981), Peru (Bianucci et al. 2018; Landini et al. 2019), Slovakia (Holec et al. 1995), Spain (Vicens and Rodríguez-Perea 2003; Mas 2009), Switzerland (Leriche 1927; Holec et al. 1995) and the USA (Purdy 1998; Kent 2018).

Genus ***Physogaleus*** Cappetta, 1980

*Type species. Trigonodus secundus* Winkler, 1876

***Physogaleus contortus*** (Gibbes, 1849)

Figure 6k, l

*Material.* One antero-lateral tooth—SNSB-BSPG 2019 III-61.

*Description.* The tooth displays a slender and distally inclined cusp (Fig. 6k, l). The cutting edges are weakly serrated along the cusp and distal heel. The distal heel is low and convex. The root is high in lingual view and low in labial view. The root/crown boundary is strongly convex in lingual view, whereas it is slightly concave in labial view. Both root lobes are short and their extremities are missing.

*Remarks.* According to Reinecke et al. (2011), the fossil record of *P. contortus* ranges from the Oligocene to the middle Miocene. Early Miocene records were reported from Italy (Marsili et al. 2007), Germany (Reinecke et al. 2011), Hungary (Kocsis 2007, as *Galeocerdo contortus*), Panama (Pimiento et al. 2013), Peru (Bianucci et al. 2018; Landini et al. 2019) and the USA (Case 1980, as *Galeocerdo contortus*; Kent 2018).

Teeth of *G. contortus* have been confused often with lower teeth of *G. aduncus*. We follow the opinion of Purdy et al. (2001) and Reinecke et al. (2011), considering the *aduncus* and *contortus* morphotypes as belonging to different genera based on substantial differences between their dental characters.

Genus ***Rhizoprionodon*** Whitley, 1929

*Type species. Carcharias (Scoliodon) crenidens* Klunzinger, 1880

***Rhizoprionodon ***sp.

Figure 6m, n

*Material.* Two antero-lateral teeth—SNSB-BSPG 2019 III-62, SNSB-BSPG 2019 III-63.

*Description.* Both teeth are abraded and part of the distal region is missing (Fig. 6m, n). The cusp is triangular and distally inclined with a flared base. The mesial cutting edge is slightly concave, whereas the distal one is straight. The distal heel is low and convex. The cutting edges are smooth along the cusp and distal heel. In lingual view, the root is high and shows a strong protuberance that is separated by a transverse nutritive groove.

*Remarks.* The genus *Rhizoprionodon* is currently represented by seven species occurring in temperate and tropical regions (Compagno 1984b). However, the genus is absent in the Mediterranean Sea. The fossil record of *Rhizoprionodon* extends back to the early Eocene (Cappetta 2012). Early Miocene records were reported from Austria (Schultz 2013), Germany (Barthelt et al. 1991; Baier et al. 2004; Reinecke et al. 2011; Pollerspöck and Straube 2017), Malta (Ward and Bonavia 2001), Switzerland (Bolliger et al. 1995) and the USA (Case 1980; Kent 2018).

According to Reinecke et al. (2011), *Rhizoprionodon* species show very similar dental characters and their identification at specific level is difficult. For this reason, we only identify our specimens at generic level.

Genus ***Isogomphodon*** Gill, 1862

*Type species. Carcharias (Prionodon) oxyrhynchus* Valenciennes, 1839

***Isogomphodon acuarius*** (Probst, 1879)

Figure 6o, p

*Material.* One lower lateral tooth—SNSB-BSPG 2019 III-64.

*Description.* The cusp is rather high and slightly distally inclined (Fig. 6o, p). The mesial and distal heels are low and mesio-distally extended. The cutting edges are smooth along the cusp and heels. The enameloid surface is completely smooth in both faces. The root is high in lingual view with a V-shaped basal face. The lobes are long with rounded extremities.

*Remarks.* The fossil record of *I. acuarius* ranges from early Miocene to the late Miocene (Cappetta 2012). Early Miocene records were reported from Costa Rica (Laurito 1999), France (Lalai, 1986), France (Cappetta 1970, as *Aprionodon acuarius*), Germany (Barthelt et al. 1991; Baier et al. 2004; Sach 2016), the USA (Case 1980), Switzerland (Bolliger et al. 1995) and Venezuela (Carrillo-Briceño et al. 2016b). The tooth described herein is similar to those from the early Miocene of Venezuela (Carrillo-Briceño et al. 2016b) and the middle Miocene of France (Vialle et al. 2011).

Family **Sphyrnidae** Gill, 1872

Genus ***Sphyrna*** Rafinesque, 1810

*Type species. Squalus zygaena* Linnaeus, 1758

***Sphyrna*** sp.

Figure 6q, r

*Material.* Seven lower lateral teeth—SNSB-BSPG 2019 III-65, SNSB-BSPG 2019 III-66 (six teeth).

*Description.* The teeth have a low and distally inclined cusp (Fig. 6q, r). The mesial cutting edges are slightly concave at their bases, but straight at their upper part. The distal heel is rather high and strongly convex. The cutting edges are smooth along the cusp and distal heel. The cusp and the distal heel are separated by a notch. The root is high and displays a vertical nutritive groove.

*Remarks.* The genus *Sphyrna* is currently represented by nine species occurring in tropical and temperate seas (Compagno 1984b). The scalloped hammerhead (*S. lewini*), the great hammerhead (*S. mokarran*), the smalleye hammerhead (*S. tudes*) and the smooth hammerhead (*S. zygaena*) are known from the present-day Mediterranean Sea (Compagno 1998). The genus *Sphyrna* extends back to the lower Oligocene (Cappetta 2012). Early Miocene records are from Austria (Schultz 1998), Colombia (Carrillo-Briceño et al. 2016a), France (Cappetta 1970), Germany (Barthelt et al. 1991; Reinecke et al. 2011), Hungary (Kordos and Solt 1984; Kocsis 2007), Malta (Ward and Bonavia 2001), Panama (Gillette 1984), Peru (Bianucci et al. 2018; Landini et al. 2019), Portugal (Antunes et al. 1981), Switzerland (Leriche, 1927), Venezuela (Carrillo-Briceño et al. 2016b) and the USA (Purdy 1998).

Reinecke et al. (2011) described *S. laevissima* and *S. integra* from the early Miocene of northern Germany. In the described lower antero-lateral teeth of *S. integra*, the crown is broader and the distal heel is straight or faintly convex. In *S. laevissima,* the crown is triangular and the distal heel is oblique. Therefore, these species show dental characters that differ from those of the specimens described here. However, as the few teeth reported here are very abraded and only represent lower teeth, we prefer to identify them only at generic level until more material is available.

Family **Scyliorhinidae** Gill, 1862

Genus ***Scyliorhinus*** de Blainville, 1816

*Type species. Squalus canicula* Linnaeus, 1758

***Scyliorhinus fossilis*** (Leriche, 1927)

Figure 7a–d

**Fig. 7:**
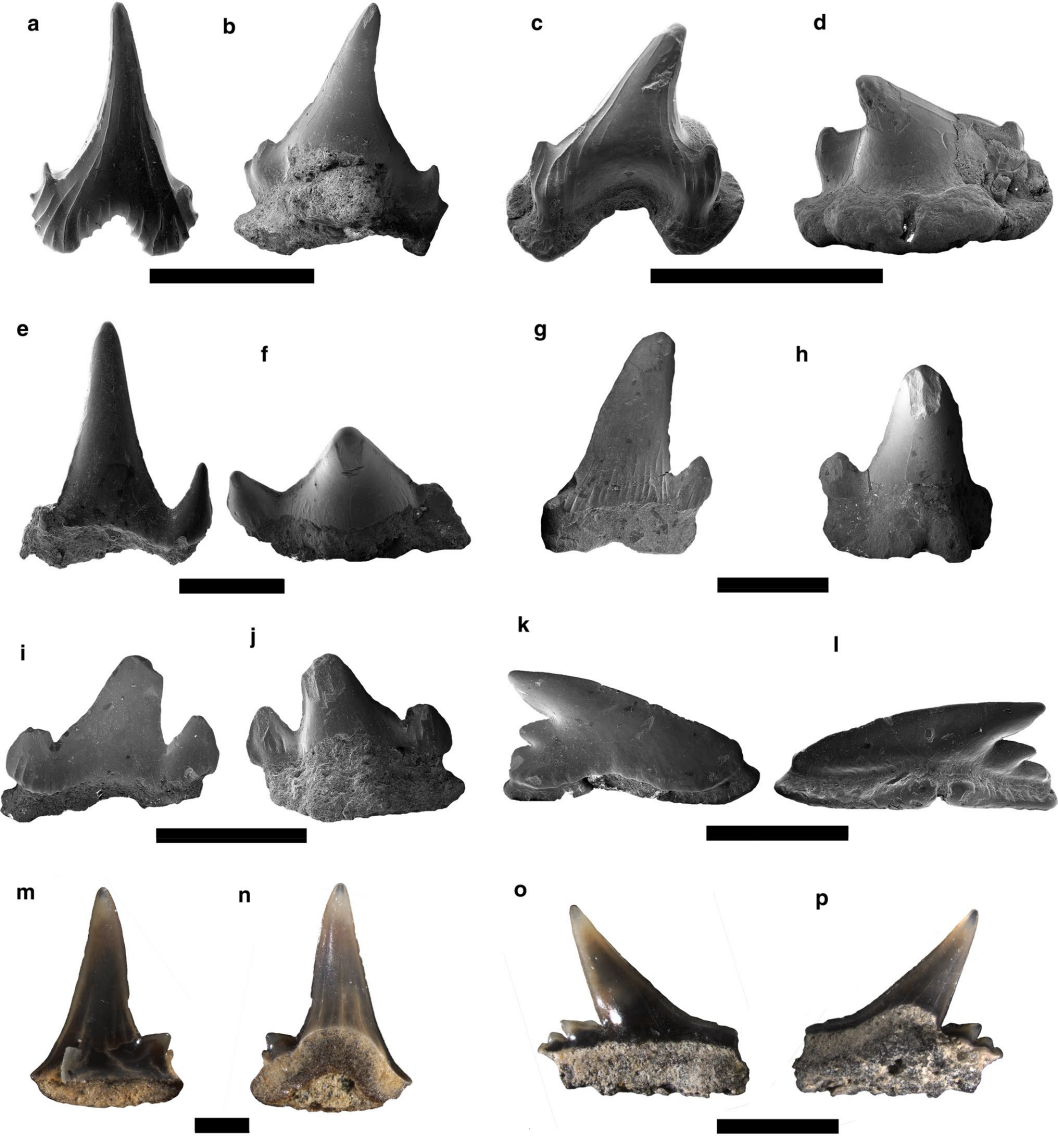
Carcharhiniformes. **a**–**d** Scyliorhinus fossilis, **a**, **b** anterior tooth, **c**, **d** lateral tooth, **e**, **f** *Scyliorhinus* sp., **g**–**j** *Pachyscyllium distans*, **g**, **h** anterior tooth, **i**, **j** lateral tooth, **k**, **l** *Chaenogaleus affinis*, upper lateral tooth, **m**–**p** *Paragaleus pulchellus*. Labial: **a**, **c**, **e**, **g**, **i**, **k**, **m**, **p**; lingual: **b**, **d**, **f**, **h**, **j**, **l**, **n**, **o**. Scale bar 1 mm

*Material.* Twelve anterior teeth—SNSB-BSPG 2019 III-67, SNSB-BSPG 2019 III-68 (11 teeth); and 12 lateral teeth—SNSB-BSPG 2019 III-69, SNSB-BSPG 2019 III-70 (8 teeth), SNSB-BSPG 2019 III-71 (3 teeth).

*Description.* The anterior teeth have a straight and slender cusp with a broader base (Fig. 7a, b). There is a pair of small, low and slightly divergent lateral cusplets, which are separated from the main cusp by a notch. A second, very incipient pair of lateral cusplets are developed at the outermost margins of the crown. Labially, strong vertical folds are present along the mesial and distal margins of the crown, extending from the crown/root boundary towards the base of the first pair of lateral cusplets. Lingually, short and closely spaced ridges are present below the lateral cusplets reaching up to the middle of the first pair of lateral cusplets and to the apex of the second pair. The cutting edges are well developed and continuous between the lateral cusplets and main cusp. The root is abraded and only the well-developed lingual protuberance is partially preserved.

In lateral teeth, the cusp is low and strongly distally inclined (Fig. 7c, d). In labial view, less pronounced folds are present along the distal and mesial margins. The root is well preserved, showing separated lobes with rounded extremities in labial view. A deep vertical nutritive groove is present on the lingual face of the root.

*Remarks. *Teeth of *S. fossilis* were reported from Miocene deposits of the Paratethys and Mediterranean regions. This species also occurs in the upper Marine Molasse of the Molasse Basin (Barthelt et al. 1991; Pfeil 1991; Reinecke et al. 2011; Schultz 2013). Additionally, it was described from the Miocene (Aquitanian to Messinian) of Switzerland, southern France, and Portugal (Leriche 1927; Antunes and Jonet 1970; Cappetta 1970, 2006; Jost et al. 2016).

The species *S. joleaudi* described by Cappetta (1970) from the Miocene of southern France represents a junior synonym of *S. fossilis*. Reinecke et al. (2011) reported the presence of *S. fossilis (*aka *S. joleaudi*) from the early Miocene of northern Germany. The teeth described here display a typical morphotype of *S. fossilis* with mesio-distally expanded crown base and several, very strong vertical folds and lateral cusplets.

***Scyliorhinus*** sp.

Figure 7e, f

*Material.* Seven lateral teeth—SNSB-BSPG 2019 III-72, SNSB-BSPG 2019 III-73 (six teeth).

*Description.* The teeth of this catshark are abraded and incompletely preserved (Fig. 7e, f). The main cusp is triangular in labial view, slightly bent distally, and lingually curved. Basally, short vertical folds are present on the lingual crown face, whereas the lingual face is smooth. The preserved cusplets are high, triangular and straight without any ornamentation. The cutting edges are completely smooth. The cusp is separated from the lateral cusplets by a deep notch. The root is heavily abraded; thus, the lingual protuberance is missing.

*Remarks. Scyliorhinus *is a diverse genus comprising 16 extant species (Froese and Pauly 2019). They are globally distributed in tropical to arctic waters, from the intertidal to the deep–sea zones (Compagno 1984b). In the Mediterranean Sea the lesser spotted dogfish (*S. canicula*), the Dumahel’s catshark (*S. duhamelii*) and the nursehound (*S. stellaris*) have been reported (Soares et al. 2019). The fossil record of *Scyliorhinus* extends back to the lower Cretaceous (Cappetta 2012). Early Miocene records were reported from Austria (Schultz 2013), Germany (Barthelt et al. 1991; Reinecke et al. 2011), Switzerland (Leriche 1927) and the USA (Case 1980).

The material described here can be unambiguously identified as *Scyliorhinus* based on the typical characters of this genus (i.e. sharp and rather slender cusp with one pair of lateral cusplets). However, due to the very abraded condition of the teeth and their incompleteness it is not possible to assign these teeth to any species known to date.

Genus ***Premontreia*** Cappetta, 1992

*Type species. Premontreia degremonti* Cappetta, 1992

***Premontreia distans ***(Probst, 1879)

Figure 7g–j

*Material.* Five anterior teeth—SNSB-BSPG 2019 III-74, SNSB-BSPG 2019 III-75 (4 teeth); and 14 lateral teeth—SNSB-BSPG 2019 III-76, SNSB-BSPG 2019 III-77 (13 teeth).

*Description.* In the anterior teeth (Fig. 7g, h), the main cusp is triangular and rather low in lingual view with one pair of lateral cusplets. The main cusp and the lateral cusplets are lingually curved. The lateral cusplets are broad and triangular. In labial view, vertical folds are present at the base of the crown. The lingual cusp face is smooth, but faint folds are developed on the lateral cusplets. The root is high in lingual view with a lingual protuberance and shallow nutritive groove. The root lobes below the lateral cusplets seem to have been very narrow as far as can be ascertained.

The lateral teeth (Fig. 7i, j) display an incomplete triangular, broad and distally inclined cusp. The lateral cusplets also are triangular and very broad. Some folds are present at the base of the lateral cusplets on both faces. The labial face of the crown overhangs the root. The crown/root boundary is concave medially and rounded at the distal and mesial regions. The root is slightly broader than the crown and is heart–shaped in basal view.

*Remarks. Premontreia distans *is very common in the Oligocene and Miocene of the North Sea basin and adjacent regions (Antunes et al. 1981; Lienau 1987; Haye et al. 2008; Reinecke et al. 2008). This taxon was originally allocated to *Scyliorhinus* (Joleaud 1912). However, Cappetta (2006) and Reinecke et al. (2008) placed this species into the extinct scyliorhinid taxon *Premontreia*. Early Miocene records were reported from France (Cappetta 1970, 1973, as *Scyliorhinus distans*), Germany (Probst 1879, as *Scyllium distans*; von Ihering 1927; Barthelt et al. 1991, as *Scyliorhinus distans*; Sachs 2016), Portugal (Antunes et al. 1981, as *Scyliorhinus distans*), Switzerland (Bolliger et al. 1995, as *Scyliorhinus distans*; Jost et al. 2016) and the USA (Case 1980). We followed the opinion of Reinecke et al (2011), considering *P. distans* as valid species based on its diagnostic characters (i.e. labial ridges at the base of the crown and the convex curvature of the mesial cutting edge). Therefore, the material described here can be unambiguously identified at species level based on the presence of those characters.

Family **Hemigaleidae** Hasse, 1879

Genus ***Chaenogaleus*** Gill, 1862

*Type species. Chaenogaleus macrostoma* (Bleeker, 1852)

***Chaenogaleus affinis ***(Probst, 1879)

Figure 7k, l

*Material.* 13 upper antero-lateral teeth—SNSB-BSPG 2019 III-78, SNSB-BSPG 2019 III-79 (2 teeth), SNSB-BSPG 2019 III-80 (10 teeth).

*Description.* The teeth show a high, broad and distally inclined cusp (Fig. 7k, l). The mesial cutting edge is convex or slightly sigmoidal in some teeth, whereas the distal cutting edges are straight or convex. The distal heel shows two to four serrations decreasing in size towards the rear. The enameloid surface displays weak folds at the base of the lingual and labial faces. The root is low and slightly wider than the crown. It shows a well-marked lingual protuberance, which is divided by a nutritive groove.

*Remarks. *The fossil species *C. affinis* ranges from the early Miocene to late Miocene (Cappetta 2012). Early Miocene records were reported from Austria (Schultz 2013), France (Cappetta 1970, as *Galeorhinus affinis*), Germany (Probst 1878, as *Galeus affinis*; von Ihering 1927; Barthelt et al. 1991; Reinecke et al. 2011; Pollerspöck and Beaury 2014; Sach 2016), Switzerland (Fischli 1930, as *Galeus affinis*; Bolliger et al. 1995; Jost et al. 2016) and the USA (Case 1980, *Galeorhinus affinis*). According to Herman et al. (2001), the teeth of the only extant species *C. macrostoma* have more elongated and slender cusps in upper antero-lateral than the fossil representative *C. affinis.* The teeth reported here also bear dental characters observed in the material from early Miocene of Northern Germany and middle Miocene of Czech Republic (Schultz et al. 2010; Reinecke et al. 2011).

Genus ***Paragaleus*** Budker, 1935

*Type species. Paragaleus gruveli* Budker, 1935

***Paragaleus pulchellus*** (Jonet, 1966)

Figure 7m–p

*Material.* One lower anterior—SNSB-BSPG 2019 III-81; and 30 lower lateral teeth—SNSB-BSPG 2019 III-82, SNSB-BSPG 2019 III-83 (19 teeth).

*Description.* The lower anterior tooth has a slender and erect cusp (Fig. 7m, n). The mesial cutting edge is concave at its base and straight at its upper part. The distal heel is short with two sharp cusplets, which are distally inclined. The enameloid surface is completely smooth. The root is incomplete; however, the lingual protuberance is well preserved.

The lower lateral tooth has a long, slender and strongly distally inclined cusp (Fig. 7o, p). The mesial cutting edge is concave, whereas the distal one is slightly convex. The distal heel is short and features three acute and distally oriented cusplets. The root is incomplete mesially and distally. The lingual protuberance is abraded, but nonetheless preserves a medial foramen.

*Remarks. *The fossil record of *P. pulchellus* ranges from the early to late Miocene (Cappetta 2012). Reinecke et al. (2011) also indicated the possible presence of *P. pulchellus* in the early Miocene of northern Germany. The taxonomic assignment of *P. pulchellus* has been debated for many years. Jonet (1966) erected *Galeorhinus pulchellus* based on the teeth from the late Miocene of Portugal. Later, Cappetta (1970) included this species within the genus *Paragaleus*. Additionally, Barthelt et al. (1991) considered *Galeorhinus tenuis* as synonym of *P. pulchellus*, a view we follow in the present study. Early Miocene records of *P. pulchellus* were reported from Austria (Schultz 2013), France (Cappetta 1970), Germany (Reinecke et al. 2011), Portugal (Antunes et al. 1981) and Venezuela (Aguilera and de Aguilera 2004).

Based on the high similarities between our teeth and those from the early Miocene of northern Germany, we identify the teeth described here as belonging to the species *P. pulchellus*.

Superorder **Batomorphii** Cappetta, 1980

Order **Myliobatiformes** Compagno, 1973

Family **Aetobatidae** Agassiz, 1858

Genus ***Aetobatus*** de Blainville, 1816

*Type species. Raja aquila* Linnaeus, 1758

***Aetobatus*** sp.

Figure 8a, b

**Fig. 8:**
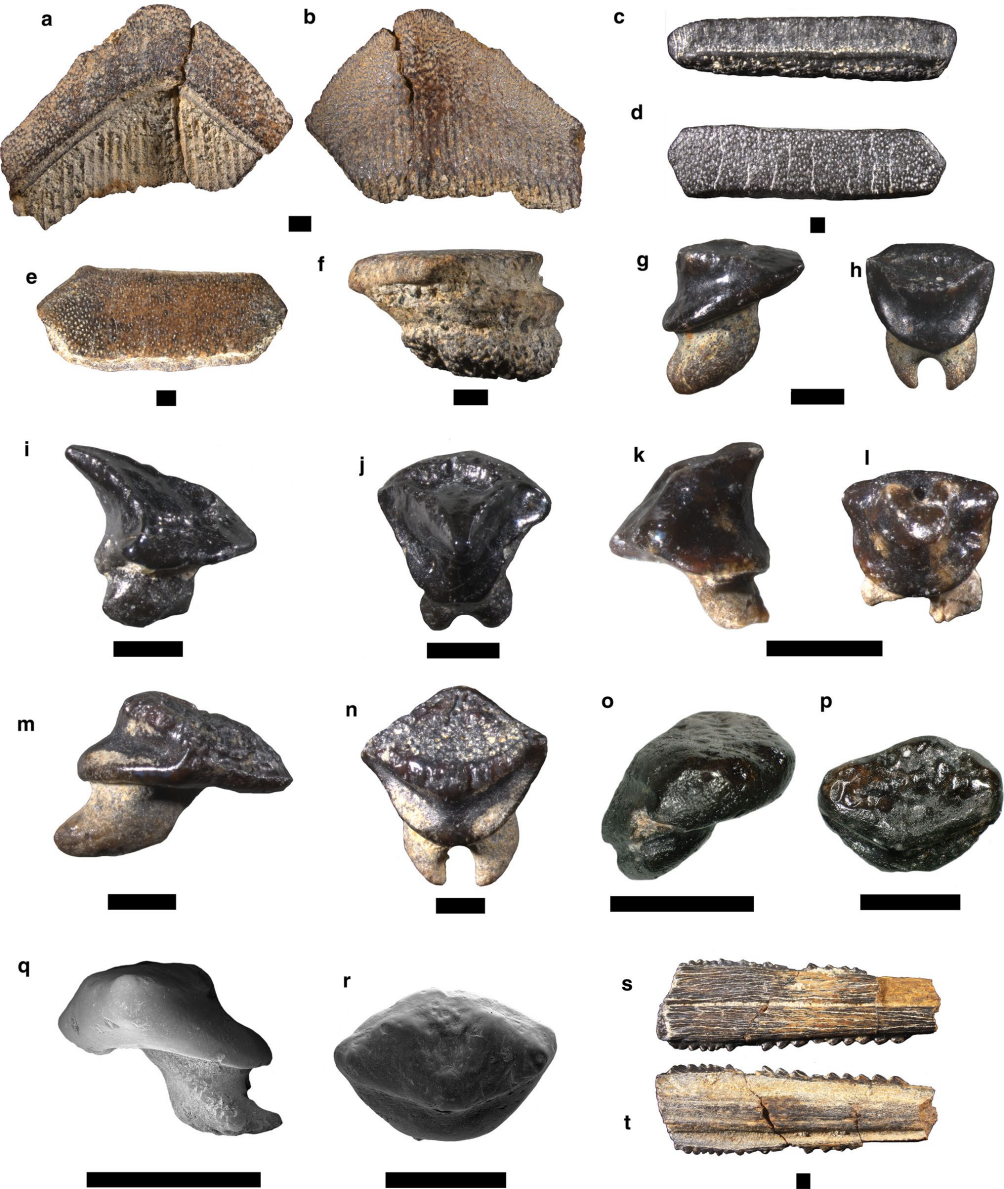
Myliobatiformes **a**, **b** *Aetobatus* sp., **c**, **d** *Myliobatis* sp., **e**, **f** *Rhinoptera* sp., **g**–**j** *Taeniurops cavernosus*, **k**, **l** *Dasyatis probsti*, **m**, **n** *Dasyatis rugosa*, **o**, **p** *Dasyatis strangulata*, **q**, **r** *Dasyatis* sp., **s**, **t** Myliobatiformes indet. Basal: **b**; dorsal: **s**; lingual: **c**, **h**, **j**, **l**, **n**; oclussal: **a**, **d**, **e**, **p**, **r**; profile: **f**, **g**, **i**, **k**, **m**, **o**, **q**; dorsal: **s**; ventral: **t**. Scale bar 1 mm

*Material.* Six lower symphysial teeth—SNSB-BSPG 2019 III-84, SNSB-BSPG 2019 III-85, SNSB-BSPG 2019 III-86 (four teeth).

*Description.* The symphyseal teeth are transversely elongated and V-shaped in occlusal view (Fig. 8a, b). The lateral edges of the symphyseal teeth form an obtuse angle. The teeth are labio-lingually thicker in the central region than in the lateral region. The crown surface is very abraded in all the examined teeth. The root vascularization is of the polyaulacorhizous type, with laminae and shallow nutritive grooves.

*Remarks. Aetobatus *is currently represented by five species: *A. flagellum*, *A. laticeps*, *A. narinari*, *A. narutobiei* and *A. oceallatus*, living in tropical and warm-temperate seas (Last et al. 2016). This genus is absent in the Mediterranean Sea. Its fossil record extends back to the upper Paleocene (Cappetta 2012). Early Miocene records were reported from Austria (Schultz 2013), Egypt (Cook et al. 2010), France (Cappetta 1970, 1973; Goedert et al. 2017), Germany (Barthelt et al. 1991; Baier et al. 2004; Reinecke et al. 2011; Sach 2016), Panamá (Gillette 1984), Portugal (Zbyszewski 1949), Slovakia (Holect et al. 1995), Switzerland (Leriche 1927; Bolliger et al. 1995) and the USA (Purdy 1998).

Our material displays the typical shape of *Aetobatus* teeth, i.e. V-shaped symphyseal teeth without any lateral teeth. Although Reinecke et al. (2011) described *A. arcuatus* from the early Miocene of northern Germany, its diagnostic characters are not very clear. According to Hovestadt and Hovestadt-Euler (2013), identification at the species level of isolated teeth only is not possible due the high intraspecific variability of dental characters. Therefore, we abstain from assigning these teeth to any species.

Family **Myliobatidae** Bonaparte, 1838

Genus ***Myliobatis*** Cuvier, 1817

*Type species. Raja aquila* Linnaeus, 1758

***Myliobatis*** sp.

Figure 8c, d

*Material.* Ten symphyseal teeth—SNSB-BSPG 2019 III-87, SNSB-BSPG 2019 III-88, SNSB-BSPG 2019 III-89 (eight teeth).

*Description.* The symphyseal teeth are very abraded and some of them are broken (Fig. 8c, d). The crown is transversely elongated with straight labial and lingual margins in occlusal view. The teeth are four to five times wider than long. All specimens have a hexagonal outline. The occlusal surface of the symphyseal teeth is smooth. The root is abraded, but it displays the typical polyaulacorhizous vascularization type.

*Remarks. *The genus *Myliobatis* is currently represented by 11 globally distributed species occurring in temperate and tropical seas (Last et al. 2016). The common eagle ray (*M. aquila*) is the only species recorded from the Mediterranean Sea (McEachran and Séret 1990). Reliable fossils of *Myliobatis* extend back to the Late Cretaceous (Claeson et al. 2010; Cappetta 2012). Early Miocene records were reported from Austria (Schultz 2013), Chile (Suarez et al. 2006), France (Cappetta 1970), Germany (Barthelt et al. 1991; Scholz and Bienert 1992; Baier et al. 2004; Reinecke et al. 2011; Pollerspöck and Beaury 2014; Sach 2016), Hungary (Kordos and Solt 1984), Panamá (Gillette 1984), Portugal (Antunes et al. 1981), Spain (Vicens and Rodríguez-Perea 2003), Switzerland (Leriche 1927; Bolliger et al. 1995), the the USA (Case 1980) and Venezuela (Aguilera and de Aguilera 2004). As in *Aetobatus*, taxonomic identification of extinct *Myliobatis* species only based on isolated teeth is extremely difficult due the high dental variation within the genus (Hovestadt and Hovestadt-Euler 2013).

Family **Rhinopteridae** Jordan and Evermann, 1896

Genus ***Rhinoptera*** Cuvier, 1829

*Type species. Myliobatis marginata* Geoffroy Saint Hilaire, 1817

***Rhinoptera*** sp.

Figure 8e, f

*Material.* Four symphyseal teeth—SNSB-BSPG 2019 III-90, SNSB-BSPG 2019 III-91 (three teeth); and three lateral teeth—SNSB-BSPG 2019 III-92, SNSB-BSPG 2019 III-93 (two teeth).

*Description.* The symphyseal teeth are broader than long with a hexagonal contour (Fig. 8e, f). In occlusal view, the teeth are straight or slightly arched lingually. The crown surface is smooth. The root shows a polyaulacorhizid vascularization type with numerous parallel laminae and nutritive grooves. The most complete specimen has 12 laminae in basal view. In profile view, the root is slightly displaced lingually and lingually separated from the crown by a bulge. The lateral tooth has a regular hexagonal outline, being less transversely enlarged than the symphyseal teeth. The crown surface is completely smooth.

*Remarks*. The genus *Rhinoptera* is currently known by eight species distributed in temperate and tropical oceans (Last et al. 2016). The Lusitanian crownose ray (*R. marginata*) is the only species reported from the Mediterranean Sea (McEachran and Séret 1990). The fossil record of *Rhinoptera* extends back into the upper Palaeocene (Cappetta 2012). Early Miocene records are from Austria (Schultz 2013), Brazil (Távora et al. 2010), France (Cappetta 1970; Goedert et al. 2017), Germany (Lutzeier 1922; Barthelt et al. 1991; Baier et al. 2004; Sach 2016), India (Mondal et al. 2009), Panamá (Gillette 1984), Portugal (Zbyszewski 1949; Antunes et al. 1981), Switzerland (Leriche 1927; Fischli 1930; Bolliger et al. 1995), the USA (Case 1980) and Venezuela (Aguilera and de Aguilera 2004).

According to Herman et al. (2000) and Cappetta (2012), the symphyseal teeth of *Rhinoptera* are longer, but less broad transversely than *Aetobatus* and *Myliobatis*. Considering these dental characters, we can assign these teeth unambiguously to the genus *Rhinoptera*. However, due to the lack of diagnostic characters, identification at specific level remains difficult.

Family **Dasyatidae** Jordan, 1888

Genus ***Taeniurops*** Garman, 1913

*Type species. Taeniura meyeni* Müller and Henle, 1841

***Taeniurops cavernosus ***(Probst, 1877)

Figure 8g–j

*Material.* 23 female teeth—SNSB-BSPG 2019 III-94, SNSB-BSPG 2019 III-95 (22 teeth); and ten male teeth—SNSB-BSPG 2019 III-96, SNSB-BSPG 2019 III-97 (8 teeth), SNSB-BSPG 2019 III-98.

*Description.* The female teeth have a rather high and acute crown, which is lingually inclined in profile view (Fig. 8g, h). The crown shows a labial and lingual visor divided by a sharp transverse crest. This crest displays some folds along the distal and mesial edges. The lower region of the labial visor is slightly convex and exhibits a reticulate ornamentation whereas the upper region is concave and smooth. The lingual visor is concave in profile view with a smooth surface. The root is high with two separated lobes.

Male teeth display a strong cuspidate and lingually oriented cusp (Fig. 8i, j). The labial visor is long with a slightly convex and ornamented lower region whereas the upper region is depressed and smooth. The transversal crest also is folded in its distal and mesial edges. The root is rather low and directed lingually with two short lobes.

*Remarks. *The fossil record of *T. cavernosus* ranges from the lower to the middle Miocene (Reinecke et al. 2011; Cappetta 2012). Early Miocene records were only reported from Germany (Probst 1877, as *Raja cavernosa*; Barthelt et al. 1991, as *Dasyatis cavernosa*; Reinecke et al. 2011; Sach 2016), Portugal (Antunes et al. 1981, as *Dasyatis cavernosa*), Switzerland (Fischli 1930, as *Trygon cavernosus*; Bolliger et al. 1995) and the USA (Case 1980). According to Cappetta (2013), the genus *Taeniurops* has been confused very often with *Dasyatis*. However, *Taeniurops* shows a distinctively depression in the labial visor which is borderer by a sharp crest, thus differing from the condition seen in *Dasyatis*. Male and female teeth reported here resemble the material described from the early Miocene of northern Germany (Reinecke et al. 2011).

Genus ***Dasyatis*** Rafinesque, 1810

*Type species. Dasyatis ujo* Rafinesque, 1810

***Dasyatis probsti ***Cappetta, 1970

Figure 8k, l

*Material.* Twelve male teeth—SNSB-BSPG 2019 III-99, SNSB-BSPG 2019 III-100 (11 teeth).

*Description.* The teeth have a cuspidate crown, which is lingually inclined (Fig. 8k, l). The labial visor is smooth and slightly convex; however, its medial region is deeply depressed. The lingual visor is also smooth and concave in profile view. The transversal crest is faintly folded. The labial margin is convex and weakly ornamented. The root is rather high and lingually oriented with two well-separated lobes.

*Remarks. Dasyatis probsti *ranges from the early to the middle Miocene (Reinecke et al. 2011). Early Miocene records were reported from France (Cappetta 1970, 1973), Germany (Reinecke et al. 2011; Pollerspöck and Beaury 2014) and Switzerland (Bolliger et al. 1995). *Dasyatis probsti* can be distinguished from *T. cavernosus* and *D*. *rugosa* by its deep depression on the labial visor and the weakly ornamented labial margin of the crown.

***Dasyatis rugosa ***Probst, 1877

Figure 8m, n

*Material.* 33 female teeth—SNSB-BSPG 2019 III-101, SNSB-BSPG 2019 III-102 (29 teeth), SNSB-BSPG 2019 III-103 (3 teeth).

*Description.* The teeth show a rather low and lingually oriented crown (Fig. 8m, n). The labial visor is convex in profile view and strongly ornamented. The lingual visor is concave in profile view with a smooth surface. In occlusal view, the labial visor is angular. The root is rather high and directed lingually with two massive lobes.

*Remarks. *The fossil record of *D. rugosa* ranges from the Oligocene to the middle Miocene (Reinecke et al. 2011). Early Miocene records were reported from Austria (Schultz 1998), France (Cappetta 1970, 1973), Germany (Probst 1877, as *Raja rugosa*; Barthelt et al. 1991; Reinecke et al. 2011; Pollerspöck and Beaury 2014; Sach 2016), Portugal (Antunes et al. 1981), Switzerland (Bolliger et al. 1995; Jost et al. 2016) and the USA (Kent 2018).

The material described here shows the diagnostic characters of teeth of *D. rugosa*, i.e. a strongly ornamented labial visor and a labial visor that appears as angular in occlusal view. These dental characters were also observed in teeth from the early Miocene of northern Germany (Reinecke et al. 2011).

***Dasyatis strangulata ***(Probst, 1877)

Figure 8o, p

*Material.* Two female teeth—SNSB-BSPG 2019 III-104, SNSB-BSPG 2019 III-105.

*Description.* The female teeth show a bulging and lingually directed crown (Fig. 8o, p). The transverse ridge is roughly pronounced, separating the labial and lingual visors. The labial visor is almost flat with a weakly reticulated surface, whereas the lingual visor is short, smooth and slightly convex in profile view. The labial margin of the crown is very thick and convex. The root is very low with two short lobes.

*Remarks. *Teeth of *D. strangulata* are very rare in the fossil record. This species ranges from the early Miocene to the Pliocene. Early Miocene records were only reported from Germany (Probst 1877, as *Raja strangulata*; Reinecke et al. 2011). *D. strangulata* can be distinguished from other species of *Dasyatis* and *Taeniurops* by the bulging crown shape and the absence of a labial depression.

***Dasyatis*** sp.

Figure 8q, r

*Material.* One female tooth—SNSB-BSPG 2019 III-106.

*Description.* The single tooth displays a rather bulging and lingually oriented cusp (Fig. 8q, r). The labial visor is weakly reticulated, whereas the lingual visor is completely smooth. In profile view, the labial visor is strongly convex and the lingual visor is concave in its medial region. In occlusal view, the crown displays a semicircular outline. The root is low with two short well-separated lobes.

*Remarks. *According to Last et al. (2016), *Dasyatis* is currently represented by five species with a global distribution. Of these, three (i.e. the marbled stingray *D. marmorata*, the common stingray *D. pastinaca* and the Tortonese’s stingray *D. tortonesi*) are currently present in the Mediterranean Sea (Cowley and Compagno 1993). The fossil record of *Dasyatis* extends back into the early Cretaceous, although most of the species might belong to different genera (Underwood et al 1999; Cappetta 2012). Early Miocene records were reported from Austria (Schultz 2013), France (Cappetta 1970), Germany (Barthelt et al. 1991; Reinecke et al. 2011), the USA (Purdy 1998) and Venezuela (Aguilera and de Aguilera 2004).

**Myliobatiformes indet.**

Figure 8s, t

*Material.* Six incomplete tail spines—SNSB-BSPG 2019 III-107, SNSB-BSPG 2019 III-108 (three incomplete tail spines), SNSB-BSPG 2019 III-109 (two incomplete tail spines).

*Description.* The six tail spines are incomplete, missing their distal and proximal portions (Fig. 8s, t). They are dorso-ventrally flattened, narrow and elongated. In dorsal view, an antero-posteriorly directed central groove and additional irregularly shaped grooves are observed. In ventral view, a central ridge is present, but weakly pronounced. Both sides of the tail spines display flat denticles that project latero-proximally.

*Remarks. *Living stingrays (Myliobatiformes) are represented by ten families (Last et al. 2016). Early Miocene records of tail spines were reported from northern Germany (Reinecke et al. 2011), Venezuela (Carrillo-Briceño et al. 2016b) and Peru (Bianucci et al. 2018; Landini et al. 2019).

According to the recent review by Hovestadt and Hovestadt-Euler (2013), in general, there are no unambiguous characters that might be useful to distinguish tail spines at genus or family level. Moreover, their morphology could also vary ontogenetically. Therefore, we prefer to keep their identification to a higher taxonomic level.

Order **Rajiformes** Berg, 1937

Family **Rajidae** de Blainville, 1816

Genus ***Raja*** Linnaeus, 1758

*Type species. Raja miraletus* Linnaeus, 1758

***Raja*** sp.

Figure 9a, d

**Fig. 9:**
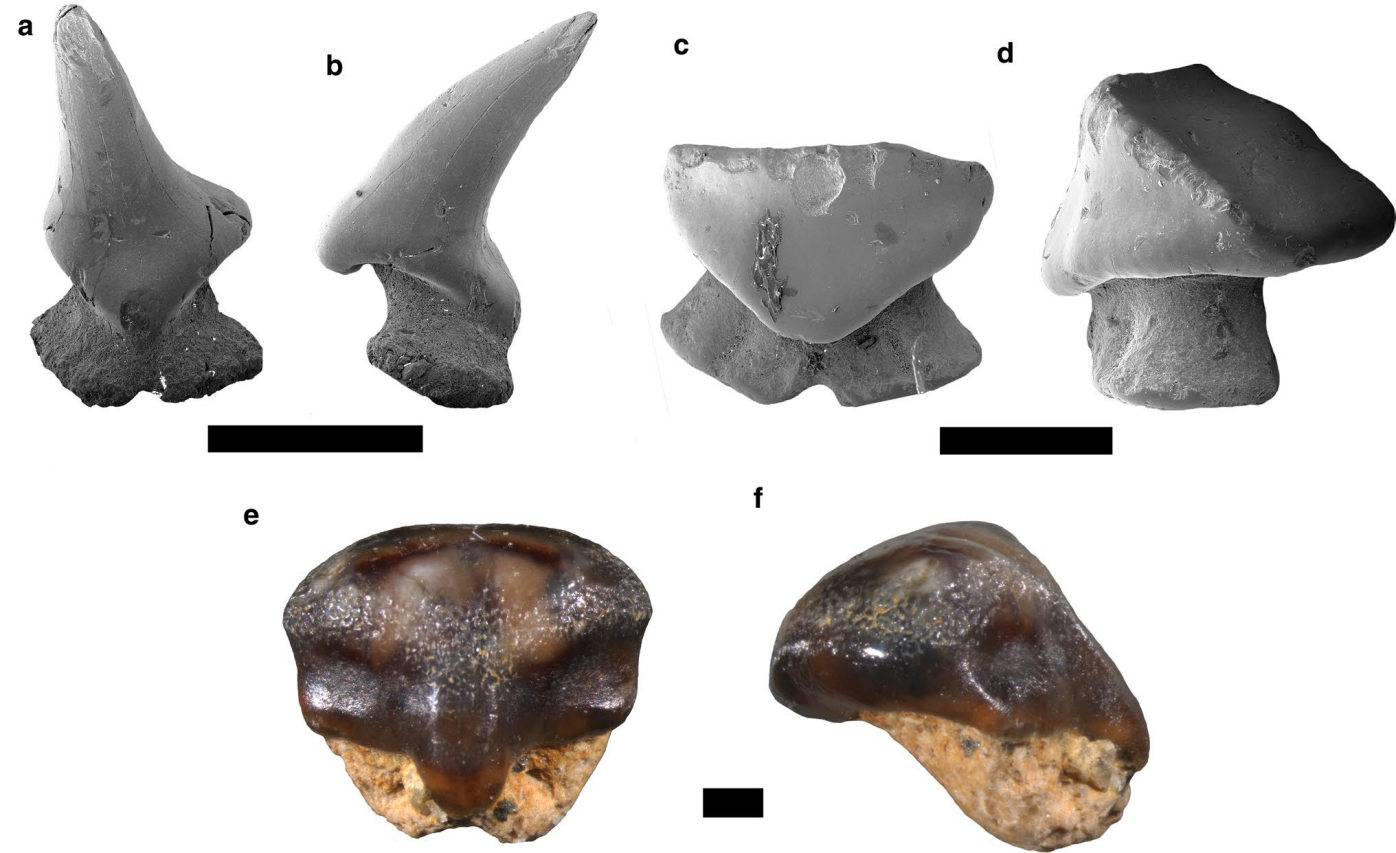
Rajiformes. **a**–**d** Raja sp., **a**, **b** male tooth, **c**, **d** female tooth, Rhinopristiformes, **e**, **f** Rhynchobatus sp. Lingual: **a**, **c**, **e**; profile: **b**, **d**, **f**. Scale bar 0.5 mm

*Material.* 17 female teeth—SNSB-BSPG 2019 III-110, SNSB-BSPG 2019 III-111 (10 teeth), SNSB-BSPG 2019 III-112 (six teeth); and 15 male teeth—SNSB-BSPG 2019 III-113, SNSB-BSPG 2019 III-114 (13 teeth), SNSB-BSPG 2019 III-115.

*Description.* Most of the teeth are very abraded and in some of them the apex is missing. The male teeth display a very high and cuspidate crown, which is lingually oriented (Fig. 9a, b). The enameloid surface is completely smooth on both the labial and lingual faces. The cutting edges are smooth along the mesial and distal edges of the cusp, and they do not reach the base of the cusp. The base of the crown displays a rounded rim with an oval shape in occlusal view. The root is low and mesio-distally expanded with two short but wide lobes. The median furrow is narrow and shallow.

The female teeth display a rounded and low crown, which is lingually oriented (Fig. 9c, d). The cutting edges are mostly smooth. They do not reach the basal rim of the crown. Some teeth display some ridges on the transverse crest in the distal and mesial edges. In profile view, the labial crown face is strongly convex whereas the lingual face is slightly convex. The root is low with two wide and short lobes.

*Remarks. *The genus *Raja* is currently represented by 16 globally distributed species (Last et al. 2016). In the Mediterranean Sea, nine species have been reported (e.g. *R. asterias* and *R. clavata*) up to now (Serena 2005). The fossil record of *Raja* extends back into the upper Cretaceous, although most of the geologically oldest species probably do not belong to the living genus (Cappetta 2012). Early Miocene records were reported from Austria (Schultz 2013), France (Cappetta 1970, 1973), Germany (Barthelt et al. 1991; Reinecke et al. 2011; Pollerspöck and Beaury 2014), Hungary (Kordos and Solt 1984), India (Sahni and Mehrotra 1981), Portugal (Antunes et al. 1981), Switzerland (Fischli 1930; Bolliger et al. 1995) and the USA (Purdy 1998; Kent 2018). Reinecke et al. (2011) described the species *Raja cecilae* and *Raja holsatica* from the early Miocene of northern Germany. However, their diagnostic characters were not described in detail. Although our material shows the general morphology of *Raja*, these dental characters are different from those described from Germany. Due to the poor preservation state, we prefer to identify them at the genus-level.

Order **Rhinopristiformes** Naylor et al., 2012

Family **Rhinidae** Müller and Henle, 1841

Genus ***Rhynchobatus*** Müller and Henle, 1837

*Type species. Rhinobatus laevis* (Bloch and Schneider, 1801)

***Rhynchobatus*** sp.

Figure 9e, f

*Material.* Five teeth—SNSB-BSPG 2019 III-116, SNSB-BSPG 2019 III-117 (four teeth).

*Description.* The teeth have a globular crown, which is wider than long (Fig. 9e, f). The crown is divided into three regions: labial, central and lingual faces. In profile view, the labial crown face is strongly convex. The central crown face is slightly depressed and weakly separated from the labial face by a transverse crest. The lingual face is oblique and slightly depressed. The crown surface is ornamented by granules around the labial and lingual faces not reaching the basal margins. The lingual uvula is wide and quite short. The root is very short, oriented lingually and divided by two lobes.

*Remarks. *The genus *Rhynchobatus* is currently represented by eight species that inhabit the Indian, western Pacific and eastern Atlantic oceans, being in turn absent in the Mediterranean Sea (Last et al. 2016). The fossil record of *Rhynchobatus* extends back into the lower Eocene (Cappetta 2012). Early Miocene records were reported from France (Cappetta 1970, 1973), Germany (Barthelt et al. 1991; Reinecke et al. 2011; Sach 2016), Malta (Ward and Bonavia 2001), Portugal (Antunes et al. 1981), Switzerland (Bolliger et al. 1995), the USA (Case 1980; Kent 2018) and Venezuela (Aguilera and de Aguilera 2004).

The teeth described herein display the typical characters of the genus *Rhynchobatus*, i.e. the oral face is divided into three regions, the enameloid is granular and the uvula is wide. A number of species of *Rhynchobatus* and *R. pristinus* have been reported from the early Miocene of Germany (Barthelt et al. 1991; Schultz 2013). However, the diagnostic characters of this species are not clear, thus, we prefer to identify it at generic level until more material is available.

## Discussion

### Taxonomic composition of Simssee fauna

**Fig. 10:**
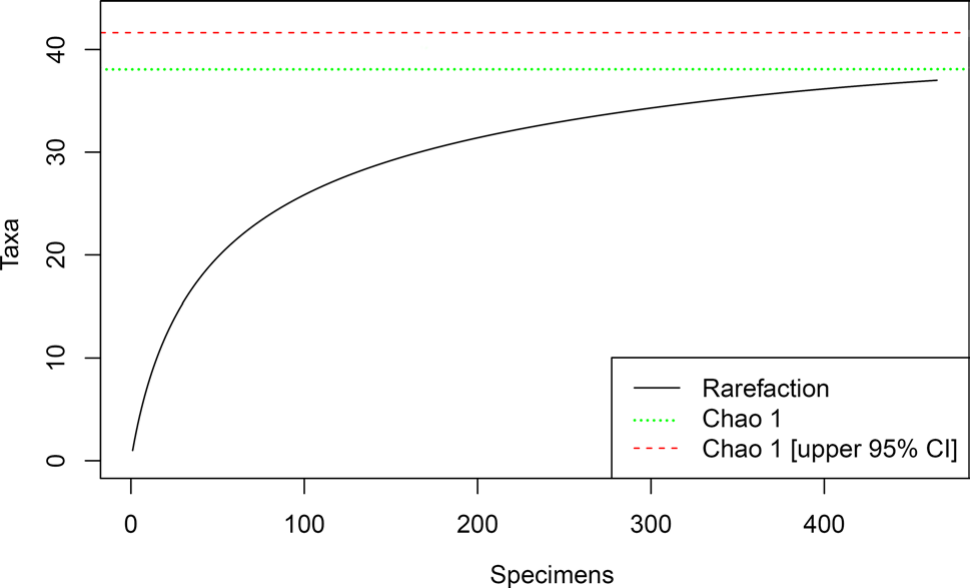
Rarefaction curve and taxonomic richness of the chondrichthyan fauna from Simssee

Elasmobranch remains are quite common in the Marine Molasse Basin of southern Germany and highlight a diversified cartilaginous fish fauna (Barthelt et al. 1991; Pollerspöck and Straube 2017; this study). Sharks, rays and skates are well represented in the fossiliferous deposits of the Achen Formation in the Simssee area. However, most of the remains are too incomplete or abraded to allow an unambiguous identification at the species level (e.g. *Myliobatis* and *Scyliorhinus*). The elasmobranch fauna from the early Miocene of Simssee/Bavaria is represented by 37 taxa (Figs. 2, 3, 4, 5, 6, 7, 8, and 9), including 26 sharks (70%, 26 out of 37 species) and 11 batoids (30%, 11 out of 37) (Table 1). The asymptotic trend of the rarefaction curve (Fig. 10) suggests that the taxonomic inventory is largely complete. In addition, the Chao 1 non-parametric estimator suggests that the completeness of the inventory would be no less than 89% (Fig. 10): indeed, the upper level confidence interval (95%) of the Chao 1 extrapolation index suggests that the total inventory would be ca. 41 taxa, i.e. 4 taxa more than those that have been observed. The sharks are mainly represented by members of the orders Carcharhiniformes (46%, 12 out of 26 shark species) and Lamniformes (23%, 6 of 26), whereas for batoids, the order Myliobatiformes is the most dominant group (82%, 9 of 11). At the family level, the carcharhinids and the dasyatids are the most diverse groups of sharks and batoids, respectively. At lower taxonomic levels, 31 genera and 20 species of early Miocene elasmobranchs were described. All the taxa described herein were previously reported from other early Miocene localities of Germany (e.g. Barthelt et al. 1991; Reinecke et al. 2011; Pollerspöck and Straube 2017). Significantly, we provided the first records of the shark species *Paragaleus pulchellus* and *Physogaleus contortus* from southern Germany. Additionally, we confirmed the presence of the rare species *Dasyatis strangulata* from Germany.

**Table 1:** Global status and present-day distribution in the Mediterranean Sea of chondrichthyans from Simsee area

Superorder	Order	Family	Taxa	Global status	Present-day distribution in the Mediterranean
Batomorphii	Myliobatiformes	Aetobatidae	*Aetobatus *sp*.*	Living	Absent
Squalomorphii	Lamniformes	Alopiidae	*Alopias exigua*	†	*A. superciliosus, A. vulpinus*
Squalomorphii	Lamniformes	Odontaspididae	*Araloselachus cuspidatus*	†	Absent
Squalomorphii	Carcharhiniformes	Carcharhinidae	*Carcharhinus priscus*	†	*C. altimus, C. brachyurus, C. brevipinna, C. falciformis, C. limbatus, C. melanopterus, C. obscurus, C. plumbeus*
Squalomorphii	Carcharhiniformes	Carcharhinidae	*Carcharhinus* sp.	Living	*C. altimus, C. brachyurus, C. brevipinna, C. falciformis, C. limbatus, C. melanopterus, C. obscurus, C. plumbeus*
Squalomorphii	Lamniformes	Odontaspididae	*Carcharias acutissimus*	†	*C. taurus*
Squalomorphii	Squaliformes	Centrophoridae	*Centrophorus granulosus*	Living	*C. granulosus, C. uyato*
Squalomorphii	Carcharhiniformes	Hemigaleidae	*Chaenogaleus affinis*	†	Absent
Squalomorphii	Hexanchiformes	Chlamydoselachidae	*Chlamydoselachus* sp.	Living	Absent
Squalomorphii	Lamniformes	Lamnidae	*Carcharodon hastalis*	†	Absent
Batomorphii	Myliobatiformes	Dasyatidae	*Dasyatis probsti*	†	*D. marmorata, D. pastinaca, D. tortonesi*
Batomorphii	Myliobatiformes	Dasyatidae	*Dasyatis rugosa*	†	*D. marmorata, D. pastinaca, D. tortonesi*
Batomorphii	Myliobatiformes	Dasyatidae	*Dasyatis* sp.	Living	*D. marmorata, D. pastinaca, D. tortonesi*
Batomorphii	Myliobatiformes	Dasyatidae	*Dasyatis strangulata*	†	*D. marmorata, D. pastinaca, D. tortonesi*
Squalomorphii	Squaliformes	Centrophoridae	Deania sp.	Living	Absent
Squalomorphii	Carcharhiniformes	Carcharhinidae	*Galeocerdo aduncus*	†	Absent
Squalomorphii	Squaliformes	Dalatiidae	*Isistius triangulus*	†	Absent
Squalomorphii	Carcharhiniformes	Carcharhinidae	*Isogomphodon acuarius*	†	Absent
Squalomorphii	Lamniformes	Mitsukurinidae	*Mitsukurina lineata*	†	*M. owstoni*
Batomorphii	Myliobatiformes	Myliobatidae	Myliobatis sp.	Living	*M. aquila*
Squalomorphii	Hexanchiformes	Hexanchidae	*Notorynchus primigenius*	†	Absent
Squalomorphii	Lamniformes	Odontaspididae	*Odontaspis molassica*	†	*O. ferox*
Squalomorphii	Carcharhiniformes	Hemigaleidae	*Paragaleus pulchellus*	†	Absent
Squalomorphii	Carcharhiniformes	Carcharhinidae	*Physogaleus contortus*	†	Absent
Squalomorphii	Carcharhiniformes	Scyliorhinidae	*Premontreia distans*	†	Absent
Squalomorphii	Pristiophoriformes	Pristiophoridae	*Pristiophorus* sp.	Living	Absent
Batomorphii	Rajiformes	Rajidae	*Raja* sp.	Living	*R. africana, R. asterias, R. brachyura, R. clavata, R. miraletus, R. montagui, R. polystigma, R. radula, R. undulata*
Batomorphii	Myliobatiformes	Myliobatidae	*Rhinoptera* sp.	Living	*R. marginata*
Squalomorphii	Carcharhiniformes	Carcharhinidae	*Rhizoprionodon* sp.	Living	Absent
Batomorphii	Rhinopristiformes	Rhinidae	*Rhynchobatus* sp.	Living	Absent
Squalomorphii	Carcharhiniformes	Scyliorhinidae	*Scyliorhinus fossilis*	†	*S. canicula, S. duhamelii, S. stellaris*
Squalomorphii	Carcharhiniformes	Scyliorhinidae	*Scyliorhinus* sp.	Living	*S. canicula, S. duhamelii, S. stellaris*
Squalomorphii	Carcharhiniformes	Sphyrnidae	*Sphyrna* sp.	Living	* S. lewini, S. mokarran, S. tudes, S. zygaena*
Squalomorphii	Squaliformes	Squalidae	*Squalus* sp.	Living	*S. acanthias*
Squalomorphii	Squatiniformes	Squatinidae	*Squatina* sp.	Living	*S. aculeata*
Batomorphii	Myliobatiformes	Dasyatidae	*Taeniurops cavernosus*	†	Absent

### Ecological traits of the identified taxa

Identifiable cartilaginous fishes from the Simssee area comprise taxa that are common elements in marine sediments of Miocene age throughout Europe (Cappetta 1970, 1973; Antunes et al. 1981; Kocsis 2007; Marsili et al. 2007; Reinecke et al. 2011; Schultz 2013). All the reported taxa are nektonic or nektobenthic organisms and some of them are able to migrate over long distances (e.g. *Alopias*, *Carcharhinus* and *Squalus*) (McFarlane and King 2003; Cartamil et al. 2010; Conrath and Musick 2010). Most of the elasmobranchs reported here are inhabitants of shallow, nearshore and littoral marine waters in warm climatic zones, according to our current knowledge about their fossil distribution, and in comparison, with modern representatives (e.g. *Aetobatus* and *Chaenogaleus*) (Last et al. 2016; Froese and Pauly 2019). This is consistent with the current distribution of cartilaginous species in the Mediterranean Sea, which are mostly distributed on the continental shelf (Froese and Pauly 2019; Ramírez-Amaro et al. 2020). However, purported deep-water sharks are also present in the Simssee area (i.e. *Chlamydoselachus, Centrophorus*, *Deania*, *Isistius*, and *Mitsukurina*). Nowadays, only few deep-water sharks can be found below 1000 m in the Mediterranean Sea (e.g. *Centrophorus granulosus* and *Hexanchus griseus*) (Sion et al. 2004). Therefore, the presence of fossil and their extant representatives indicates that the sediments of the Simssee area were deposited in shallow-to-deep shelf environments. According to Kroh (2007), the echinoderm fauna from the early Miocene of the Central Paratethys inhabited shallow and deep-water environments. The co-presence of elasmobranchs inhabiting shallow and deep environments was also reported from other lower Miocene, Pliocene and Pleistocene European localities (Kocsis 2007; Marsili 2007; Fulgosi et al. 2009; Reinecke et al. 2011; Pollerspöck and Straube 2017).

### Paleobiogeographic dynamics

The elasmobranch fauna described here experienced paleobiogeographic changes from the early Miocene to the recent (Table 1). At genus level, 10% of the recognised genera (3 out of 31) are globally extinct (*Araloselachus, Physogaleus* and *Premontreia*). Comparing the presence of the survived genera in the Mediterranean Sea today, two biogeographic dynamics are observed. Fifty percent of the living genera (14 out of 28) are absent in the Mediterranean Sea (*Aetobatus, Chaeonagelus*, *Chlamydoselachus*, *Carcharodon*, *Deania*, *Galeocerdo*, *Isitius, Isogomphodon*, *Notorynchus*, *Paragaleus*, *Pristiophorus*, *Rhizoprionodon*, *Rhynchobatus* and *Taeniurops*), whereas 50% (14 of 28) are still present being represented by at least one species. For instance, the genera *Squalus* and *Myliobatis* are currently represented in the Mediterranean Sea by the picked dogfish shark *S. acanthias* and the common eagle ray *M. aquila* (Compagno 1988; Last et al. 2016). At the species level, all the recognized species but one (i.e. *Centrophorus granulosus*) are globally extinct. Although most of the recognized species have disappeared from the Mediterranean Sea, different biogeographic dynamics are observed by considering replacement by congeneric species. Fifty-five percent (11 of 20) of the extinct species were not replaced by congeneric species living today in the Mediterranean Sea. On the contrary, 45% (9 of 20) of the extinct species have been substituted by at least one congeneric species living in the present-day Mediterranean Sea. For instance, *Alopias exigua* is globally extinct, but the extant thresher shark *Alopias vulpinus* currently occurs in the Mediterranean Sea (Compagno et al. 2005). All these biogeographic dynamics could have been influenced by intense tectonic, climatic and oceanographic events during the early Miocene of Europe (Rögl 1999). According to Kroh (2007), the climatic and oceanographic changes (i.e. drop of temperature and sea level changes) were the major factors controlling the distribution of echinoderm faunas during the early Miocene of the Central Paratethys, and may have also affected elasmobranchs. This idea was previously hypothesized as a possible cause of chondrichthyan distributional changes in South American localities during the Neogene (Long 1993; Cione et al. 2007; Carrillo-Briceño et al. 2013; Villafaña 2015; Partarrieu et al. 2018; Villafaña and Rivadeneira, 2014, 2018; Villafaña et al. 2019).

### Faunal comparison during the early Miocene

**Fig. 11:**
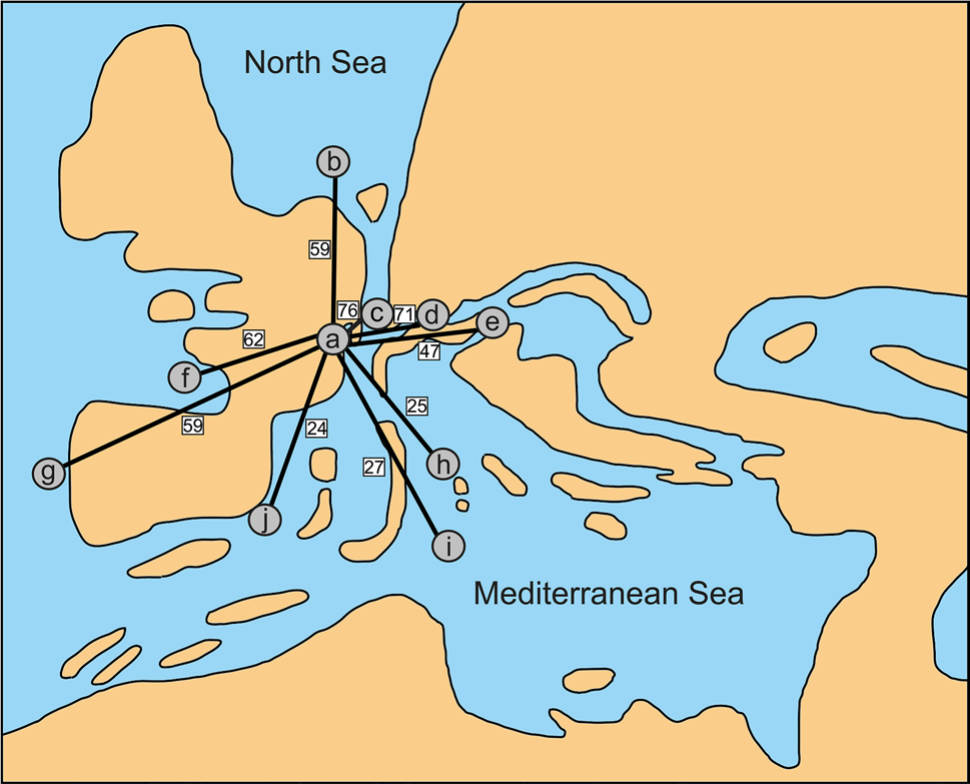
Paleogeographic distribution and faunal similarity between the Simssee fauna and others early Miocene faunas. Simssee (**a**), Northern Germany (**b**), Switzerland (**c**), Austria (**d**), Slovakia (**e**), France (**f**), Portugal (**g**), Italy (**h**), Malta (**i**) and Spain (**j**). Map based on Rögl (1999)

According to our faunal comparison, the fauna from the Simssee area was more similar to closely adjacent localities in Europe rather than to other localities (Fig. 11 and Table 2). The most similar faunas are from Switzerland (76%), Austria (71%), France (62%), North Germany (59%) and Portugal (59%). These high similarities could be related to the shorter distances and connection between the localities. During the Ottnangian, the western and Central Paratethys were connected through the Rhine Graben (Rögl 1999). According to Kocsis (2007), the presence of deep-water sharks such as *Mitsukurina* and *Isistius* should be the evidence of large and connected open water surfaces, with deeper sea basin during the Eggenburgian–Ottnangian stages. According to Pollerspöck and Straube (2017), the diversity of fishes from the Paratethys was shaped by immigration of taxa from other marine ecosystems and favoured by oceanographic variables such as salinity and oxygen contents. Additionally, dispersal of some sharks could be also possible into freshwater environment (Kocsis et al. 2007). Therefore, the seaway passages could have favoured the connection of elasmobranch faunas from different localities and explain their similarities. On the contrary, the faunas with the lowest similarities were from Colombia (20%), Panamá (23%), Spain (24%) and Brazil (24%), possibly reflecting to the long distances between the localities. Despite the apparent connection of Italy, Malta, and Spain with southern Germany during the Ottnangian (Rögl 1999), their lower similarity could be affected by sampling biases (i.e. incompleteness of the taxonomic inventories). However, areas where sampling is expected to be high (e.g. the Pacific coast of USA, Australia) also show low similarity values, due to the relatively low generic richness. In the case of India (29%), the seaway passage between the Indo-Pacific and the Paratethys was closed during the Ottnangian, probably explaining its lower similarity to the fauna from Simssee.

**Table 2:** Faunal similarity between the Simsee fauna and other early Miocene faunas

Country	Combined localities	Stages	Number of genera	Shared genera	Dice’s Similarity	References
Switzerland	Benken, Zurich; Lucerne, Gallen	Burdigalian	41	27	0.76	Fischli (1930), Bolliger et al. (1995) and Jost et al. (2016)
Austria	Eggenburg, Maigen, Kletzenmarkt, Oberösterreich, Plesching, Wallern	Eggenburgian–Ottnangian	46	27	0.71	Schultz (2013) and Pollerspock et al. (2018)
France	Bordeaux, Breyra Valley, Gironde, Léognan, Montpellier, Saucats	Aquitanian–Burdigalian	41	22	0.62	Chevalier (1961), Cappetta (1970), Cahuzac et al. (2007) and Goedert et al. (2017)
North Germany	Werder, Uesen	Burdigalian	38	20	0.59	Reinecke et al. (2011)
Portugal	Quarry Quinta das Pedreiras, do Narigao, da Noiva	Aquitanian–Burdigalian	28	17	0.59	Zbyszewski (1949)
USA-east	Calvert County, Cumberland County, Harbourtown Marina	Aquitanian–Burdigalian	33	17	0.54	Cope (1870), Eastman (1904) and Case (1980)
Slovakia	Cerova, Lieskove	Burdigalian	30	14	0.47	Underwood and Schlogl (2013)
Hungary	Ipolytarnoc	Burdigalian	20	10	0.40	Kordos and Solt (1984) and Kocsis (2007)
Chile	La Boca, Punta Perro	Aquitanian–Burdigalian	12	8	0.38	Suarez et al. (2006)
India	Baripada, Gogha	Aquitanian–Burdigalian	18	7	0.29	Eames (1937) and Sahni (1979)
USA-west	Point Arena, Jewett Sand	Aquitanian	12	6	0.29	Philips and Welton (1976) and Welton (1981)
Malta	Ras ir-Reqqa, Bahrija, Rdum ii-Vigarju	Aquitanian–Burdigalian	15	6	0.27	Ward and Bonavia (2001)
Australia	Strathalbyn	Aquitanian–Burdigalian	10	5	0.25	Pledge (1967)
Italy	Montagna della Maiella	Burdigalian	10	5	0.25	Marsili et al. (2007)
Brazil	Colonia Pedro Teixeira, Jazida, Praia de Fortalezinha	Aquitanian–Burdigalian	11	5	0.24	Araujo-Tavora et al. (2010)
Spain	Elche, Cala Sant Vicenç	Burdigalian	11	5	0.24	Mendiola (1996, 1997), Vicens and Rodriguez (2003) and Mas (2009)
Panama	Cartagena, Las Cascadas, Lirio, Pacuare de Tres Equis	Aquitanian–Burdigalian	13	5	0.23	Pimiento et al. (2013) and Laurito et al. (2014)
Colombia	Arroyo Uitpa	Aquitanian	11	4	0.20	Carrillo-Briceño et al. (2016a, b)
